# Evolution of physical linkage between loci controlling ecological traits and mating preferences

**DOI:** 10.1111/jeb.14105

**Published:** 2022-10-05

**Authors:** Tarryn Schuldiner‐Harpaz, Richard M. Merrill, Chris D. Jiggins

**Affiliations:** ^1^ Department of Zoology University of Cambridge Cambridge UK; ^2^ Ludwig‐Maximilians‐Universität Munich Germany

**Keywords:** divergent selection, genetic coupling, linkage disequilibrium, physical linkage, reproductive isolation, speciation

## Abstract

Coupling of multiple barriers to gene‐flow, such as divergent local adaptation and reproductive isolation, facilitates speciation. However, alleles at loci that contribute to barrier effects can be dissociated by recombination. Models of linkage between diverging alleles often consider elements that reduce recombination, such as chromosomal inversions and alleles that modify recombination rate between existing loci. In contrast, here, we consider the evolution of linkage due to the close proximity of loci on the same chromosome. Examples of such physical linkage exist in several species, but in other cases, strong associations are maintained without physical linkage. We use an individual‐based model to study the conditions under which the physical linkage between loci controlling ecological traits and mating preferences might be expected to evolve. We modelled a single locus controlling an ecological trait that acts also as a mating cue. Mating preferences are controlled by multiple loci, formed by mutations that are randomly placed in the “genome”, within varying distances from the ecological trait locus, allowing us to examine which genomic architectures spread across the population. Our model reveals that stronger physical linkage is favoured when mating preferences and selection are weaker. Under such conditions mating among divergent phenotypes is more frequent, and matching ecological trait and mating preference alleles are more likely to become dissociated by recombination, favouring the evolution of genetic linkage. While most theoretical studies on clustering of divergent loci focus on how physical linkage influences speciation, we show how physical linkage itself can arise, establishing conditions that can favour speciation.

## Graphical abstract

Mating preference loci spread more easily across the population when they are within proximity to the trait locus.

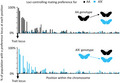

## 1 INTRODUCTION

A central element of speciation with gene‐flow is the coupling of multiple barrier effects, which together generate strong reproductive isolation (Butlin and Smadja, 2018; Felsenstein, 1981; Maynard Smith, 1966; Smadja and Butlin, 2011). For example, divergence in female mating preferences based on a male trait that is under divergent selection can suppress gene flow, leading to speciation. For such coupling to occur, alleles involved in divergence must be in linkage disequilibrium, defined as the non‐random association of alleles at different loci (Felsenstein, 1981; Lewontin & Kojima, 1960). In a seminal theoretical paper, Felsenstein (1981) argued that when diverging populations remain in contact, recombination will disrupt linkage disequilibrium between alleles involved in divergence. He, therefore, argued that recombination is a fundamental force impeding speciation.

However, the dissociating force of recombination can be avoided or reduced in many ways. For example, when a single locus controls more than one barrier effect either by pleiotropy (Barton et al., 2007; Maynard‐Smith, 1966), or in the case of ‘magic traits’, i.e. traits that are under divergent selection and also act as mating cues (Gavrilets, 2004). The latter are now considered widespread (Servedio et al., 2011). However, if barriers to gene flow are controlled by separate genetic elements, linkage disequilibrium can be maintained if the relevant loci are either within regions of the genome with reduced recombination, such as sex chromosomes and chromosomal inversions (Butlin, 2005; Kirkpatrick and Barton, 2006; Kozak et al., 2017; Ortiz‐Barrientos et al., 2016; Trickett and Butlin, 1994), or are physically linked, i.e, in close proximity on the same chromosome (Maynard Smith, 1977; Ortiz‐Barrientos et al., 2016).

Genomic architectures that alleviate the dissociating force of recombination have been studied extensively in the context of local adaptation and speciation. Theoretical studies of linkage have largely focused on the influence of elements that suppress recombination on the likelihood and progress of speciation (Feder et al., 2012; Flaxman et al., 2014; Servedio and Bürger, 2018), whereas the evolution of such elements per se has received much less attention (Yeaman and Whitlock, 2011). The notion that linkage intensity can be subject to natural selection was introduced long ago (Nei, 1967). Where selection favours two or more phenotypic optima with intermediate, maladaptive phenotypes, we expect stronger linkage to evolve between alleles that contribute to local adaptation and those that contribute to reproductive isolation. In such a case, we might also expect selection to favour genomic architectures that reduce recombination between the relevant loci. For example, Trickett and Butlin (1994) extended Felsenstein's original model (1981) to include a recombination suppressor that reduces recombination between a locus under divergent selection and a locus with an influence on assortative mating (i.e., more frequent mating among individuals with similar phenotypes; Otto et al., 2008). They found that the suppressor could spread across the population, facilitating the establishment of disequilibrium and increasing reproductive isolation. Other models have explored changes in the rate of recombination between diverging loci caused either by chromosomal rearrangements, such as inversions, that capture locally favoured combinations of genes (Kirkpatrick and Barton, 2006; Feder et al., 2011) or as modifier alleles that alter recombination rates between separate loci (Charlesworth and Charlesworth, 1979; Nei, 1967; Pylkov et al., 1998). These studies show that under certain conditions, elements that reduce recombination between diverging loci can spread across the population, thereby facilitating speciation.

However, in contrast to models that address elements such as chromosomal rearrangements and modifier alleles that reduce recombination between existing loci, here, we consider the evolution of linkage due to the close proximity of loci on the same chromosome. Specifically, we focus on the physical linkage between loci that control traits under divergent selection (hereafter ‘ecological traits’) and those that contribute to mating preferences (i.e., a ‘preference/trait’ system, as defined by Kopp et al., 2018). There are a number of empirical examples of physical linkage between loci controlling ecological traits and mating preferences. For example, warning patterns in the sympatric butterfly species *Heliconius melpomene* and *H. cydno* are under divergent selection (Merrill et al., 2012). A major locus underlying differences between these species in matin g preference is in close proximity (~1.2 cM) to a key colour pattern gene (Merrill et al., 2019; Rossi et al., 2020). Physical linkage has also been reported between loci controlling body size and shape, which are known to be under divergent selection, and female mate choice in threespine sticklebacks (*Gasterosteus aculeatus*; Bay et al., 2017), and between loci controlling host plant choice and performance on hosts in pea aphids (*Acyrthosiphon pisum pisum*; Hawthorne and Via, 2001).

The mechanism by which loci controlling ecological traits and mating preferences may become physically linked remains unknown. In the context of supergene evolution, there has been an ongoing theoretical debate as to whether loci must be physically linked at the outset or whether they can be brought into closer physical association by genomic rearrangements (Charlesworth and Charlesworth, 1976; Joron et al., 2011). Models of local adaptation show that clustering of locally favourable alleles may arise due to the transposition of loci from one position on a chromosome to another (Ortiz‐Barrientos et al., 2016; Yeaman, 2013) or by changes in gene order following chromosomal rearrangements (Ortiz‐Barrientos et al., 2016). However, for example, in the sister butterfly species mentioned earlier, *Heliconius melpomene* and *H. cydno*, there is no evidence of transposition around colour pattern genes or chromosomal inversions that might be involved in maintaining species barriers (Dasmahapatra et al., 2012; Davey et al., 2017). If ecological trait and mating preference loci are not brought together by genomic rearrangements, the physical linkage may evolve if existing genes that happen to be within proximity to ecological trait genes are coopted to control mating behaviours. Genomic studies have shown that, in many cases, mating preference is a polygenic trait controlled by multiple loci (Bakker and Pomiankowski, 1995; Bay et al., 2017; Merrill et al., 2019). In such cases, loci involved in mating preferences may arise at various locations within the genome, and selection is expected to favour those that arise within close proximity to loci controlling the ecological trait. This is analogous to the mechanism proposed by Charlesworth and Charlesworth (1976) for the evolution of supergenes underlying mimicry in butterflies, also termed Turner's sieve (Jiggins, 2017; Turner, 1977, 1987).

However, linkage disequilibrium can also be maintained between ecological and mating trait alleles by a combination of strong selection and assortative mating, even in the absence of physical linkage. For example, hamlets (*Hypoplectrus*) differ most notably in pigmentation, which is under divergent selection for crypsis and mimicry, and also mate assortatively based on visual cues. Vision and pigmentation genes in hamlets are within linkage disequilibrium despite being distant from each other or even on separate chromosomes (Hench et al., 2019). This raises the question of which evolutionary forces influence the development of physical linkage between loci controlling ecological traits and mating preferences.

To explore which conditions might favour the evolution of physical linkage, we used an individual‐based simulation model. This approach allows observing how genomic architectures at the individual level spread across the population and influence reproductive isolation at the population level. We modelled a single locus controlling an ecological trait that acts also as a mating cue (a “magic trait”, Gavrilets, 2004). Mating preferences are caused by mutations that are randomly placed in the “genome”, within varying distances from the ecological trait locus (i.e., within varying strengths of physical linkage). While we expect strong mating preferences to arise due to the accumulation of multiple preference mutations, we are not so much interested in the number of loci underlying mating preference but rather use this polygenic architecture as a tool to study under which conditions physically linked loci are favoured. Specifically, the model was designed to explore the influence of selection strength and mating preference strength on the evolution of physical linkage between loci controlling ecological traits and mating preferences. To the best of our knowledge, this is the first theoretical study to explore (i) forces that shape the relative location of trait and preference loci in the genome and (ii) evolution of physical linkage by “filtering” of preference loci that arise in proximity to ecological trait loci.

## 2 METHODS

### 2.1 Model description

The model was implemented in NetLogo 6.1.1 (Wilensky, 1999). The model NetLogo file, including the source code, is available at the following link: http://ccl.northwestern.edu/netlogo/models/community/physical_linkage_one_trait_locus. The model description follows the ODD (Overview, Design concepts and Details) protocol for individual‐based models (Grimm et al., 2006, 2010). Here, we provide a summary of the model description. The complete ODD is available in Appendix S1.

The model consists of 10 000 individuals with an even male:female ratio. Individuals are diploid with a single pair of homologous chromosomes, each containing a sequence of 100 loci, defined together as the “genome”. The first locus controls an ecological trait, for which there are two possible alleles, with an initially equal frequency in the population: A or A′. The alleles are codominant and therefore, the three possible genotypes produce three separate phenotypes: AA, A′A′ homozygotes or an intermediate, heterozygote AA′ phenotype. The ecological trait is subject to selection but also serves as a mating cue and can therefore be considered a “magic trait” (Gavrilets, 2004). The remaining 99 loci on each chromosome can potentially be subject to mutations that cause a preference to mate with either AA or A′A′ phenotypes (preference alleles) or have no influence on mating preference (neutral alleles), for comparison. We hereafter refer to loci at which a mutation has occurred as preference loci or neutral loci, respectively. The mutations occur at an equal probability, in one percent of individuals every generation, at a single, randomly chosen locus.

The modelled environment comprises two habitats, such that selection favours the AA phenotype in one habitat and A′A′ phenotype in the other. The habitats are equally maladaptive for the heterozygote AA′ phenotype and for the non‐locally adapted homozygote phenotype in each habitat. The adaptive allele is thus effectively recessive within each habitat, even though there are three distinct phenotypes, AA, AA′ and A′A′ (that play a role in mate choice, see below). Selection is modelled to reflect a scenario of two separate phenotypic optima with sub‐optimal hybrid phenotypes. Habitats are represented in the model in a non‐spatial manner. Individuals remain in the habitat to which they are initially assigned, except while mating.

Each generation, several stages are executed in the following order:

#### 2.1.1 Formation of mating pairs

Mate choice is made by the females, who are randomly paired with males from both habitats, reflecting a scenario of unrestricted movement of males between habitats when searching for a mate. A female will mate with a male with whom she is paired at a probability that depends on the strength of her preference for his phenotype (explained in detail below). If a female decides not to mate, she is paired sequentially with a maximum of 10 random males until she mates. If she does not mate with the tenth male with which she is paired, she does not reproduce. Limiting the number of rejections to 10 avoids simulations from running endlessly while maintaining negligible costs of female mating preference (Kopp and Hermisson, 2008; Schneider and Bürger, 2006). See Appendix S2.4 for details on the sensitivity of model results to changes in the maximum number of males with which a female is paired. Each individual is allowed to mate once. Allowing males to mate with multiple females did not influence the general trends in simulation results (see Appendix S3.1 for details).

**FIGURE 1:**
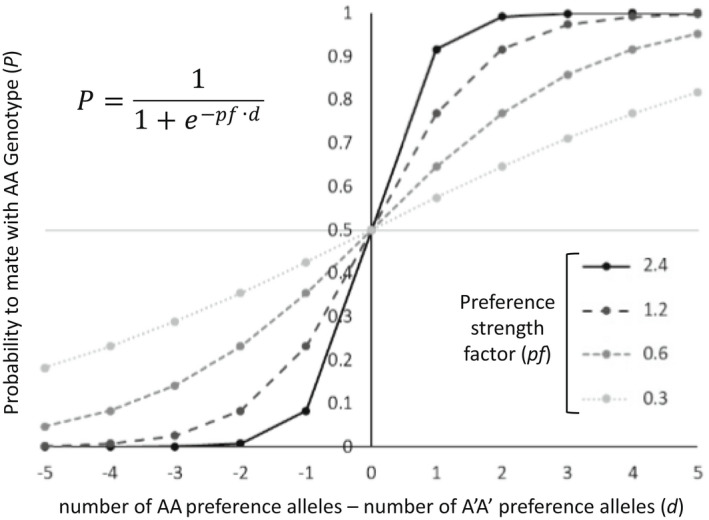
Modelling mating preference strength. The probability to mate (*p*) depends on the number of preference alleles and on the preference strength factors (*pf*). For stronger *pf* values, mating preferences develop at a higher rate. The graph presents the probability to mate with AA genotyped individuals. The probability to mate with A′A′ genotyped individuals equals 1 − *p*. A grey horizontal line marks the probability to mate with a heterozygote (AA′ genotype), which does not depend on the number of preference loci and is always intermediate between the probabilities to mate with either of the homozygotes (AA and A′A′ genotype).

The probability of a female to mate with a male with which she is paired is described by the following equations (Figure 1):

$$P_{A}=\frac{1}{1+e^{-d∙\mathrm{pf}}}$$

$$P_{A\prime }=1-P_{A}$$

Where *P*_*A*_ and *P*_*A′*_ are the probabilities to mate with a male of phenotype AA and A′A′, respectively. *d* is the difference between the number of AA preference alleles and the number of A′A′ preference alleles in the female's genome. The higher the number of AA preference alleles, compared to A′A′ preference alleles, the larger the probability to mate with an AA phenotyped male, and vice versa. A preference strength factor (*pf*) determines how strong the contribution of each additional preference allele is.

The probability to mate with a heterozygote male (AA′ phenotype) is constant for all individuals and equals 0.5. This represents a case in which hybrids, represented by the heterozygotes in this model, exhibit intermediate phenotypes which are partially attractive to individuals who prefer to mate with one of the distinct phenotypes, represented as homozygotes in this model. Robustness of model results to changes in this assumption are detailed in Appendix S3.2.

The preference strength factor (*pf*) is used to control the rate at which strong mating preferences accumulate across the population. Varying the value of *pf* allows testing the influence of mating preference strength on the development of physical linkage between ecological trait and preference loci.

Directional selection on the ecological trait locus caused simply by the existence of mating preferences is notable in our model only if the divergent selection is not strong enough to maintain the stable coexistence of the two homozygote phenotypes. Under such a scenario, slight differences in the frequencies of the homozygote phenotypes will cause a disadvantage to the less frequent phenotype in finding a mating partner, resulting in a continued decline in frequency and rapid collapse of the less frequent phenotype. (see Appendix S2.1 for further detail).

#### 2.1.2 Reproduction

Only individuals who have found a mating partner in the previous procedure will reproduce. Each mating pair produces four offspring, two males and two females. The number of offspring was chosen to keep population size above carrying capacity before selection and density dependent regulation take place to avoid population collapse.

Each offspring receives one paternal and one maternal chromosome, randomly chosen from the two chromosomes of each parent. Offspring are assigned the same habitat as their mother to ensure that the mating choice has a direct influence on the survival of offspring and is, therefore, subject to natural selection. Following reproduction, the parent generation dies.

#### 2.1.3 Offspring recombination and mutation

Each offspring “genome” undergoes recombination at one, randomly chosen cross‐over point within the chromosomes. The content of the “genome” sequence, after the cross‐over point, is exchanged between the two homologous chromosomes. Recombination does not change the offspring genotype or number of preference alleles, and therefore, has no influence on the offspring itself, and can be seen as representing recombination in the offspring gametes, as preparation for mating. See Appendix S3.3 for the robustness of model results to changes in the number of recombination events per parent.

After recombination is completed, mutations are added, causing mating preference for either AA or A′A′ phenotypes (preference alleles), or having no influence on mating preferences (neutral alleles). The neutral alleles are added as a control to explore the basic level of physical linkage expected between the ecological trait locus and random mutations with no influence on preference. The mutations occur in 100 randomly selected individuals (one percent of the population) at a single locus, randomly selected from one of the two chromosomes. The three possible mutations occur at equal probabilities, at a randomly chosen location within the “genome”, excluding the first locus, which controls the ecological trait. Mutation may occur, at a low probability, twice at the same locus. Therefore, changes in preference alleles are possible, as are back mutations, if a neutral mutation occurs at a locus at which a preference mutation occurred previously. As mutation occurs at a single locus, chosen from two chromosomes, in one percent of individuals, the mutation rate per locus is $5∙10^{-5}$. See Appendix S2.3 for details on the sensitivity of model results to changes in mutation rate, and Appendix S3.4 for the robustness of model results to changes in the level at which mutation rate is implemented (at the population level or at the single locus level).

#### 2.1.4 Ecological selection

Individuals that are not favoured in each of the habitats are subject to selection, i.e., any phenotype other than AA in one habitat and other than A′A′ in the second habitat. The strength of selection is determined by a selection coefficient (*s*), defined as the proportion of maladapted individuals that die each generation as a result of selection. Robustness of model results to changes in the relative selection level against heterozygote, intermediate, phenotypes are detailed in Appendix S3.5.

#### 2.1.5 Density dependent regulation

Each of the two habitats has a carrying capacity of 5000 individuals. Density dependent regulation is therefore implemented by randomly selecting 5000 surviving individuals in each habitat. Separate density dependent regulation of population size in each habitat is based on the assumption that individuals depend on separate, limited, resources in each habitat.

Time steps in the model correspond to discrete, non‐overlapping generations. Each simulation was run for 3000 generations, well after assortative mating was established and each of the two habitats was populated by a single homozygote phenotype.

The sensitivity of model results to changes in parameter values (i.e., selection coefficient, preference strength factor, mutation rate, maximum attempts to find a mate, genome length and carrying capacity), as well as the robustness of results in response to changes in model assumptions, are described in the Appendices S2 and S3.

Furthermore, as an alternative, we developed an additional model in which the ecological trait is controlled by two separate loci, which arguably better reflects the genetic architecture of incompatibilities observed in hybrids between divergent taxa. However, results from the two‐locus model were similar to the original one‐locus model, as shown in detail in Appendix S3.6.

### 2.2 Simulation analyses

#### 2.2.1 Testing the influence of selection and mating preference strength on the development of assortative mating and physical linkage

To test the effect of the strength of selection and mating preferences on the development of (i) assortative mating and (ii) physical linkage between ecological trait and preference loci, 20 simulations were carried out for every combination of selection coefficient (*s* = 0.5, 0.6, 0.7 and 0.8) and preference strength factor (*pf* = 0.3, 0.6, 1.2 and 2.4). The maximum *pf* value was set to 2.4 because for this value, an excess of only one preference allele for a specific phenotype is needed for there to be a probability of over 0.9 to mate with an individual of the preferred phenotype (Figure 1). Therefore, the difference in preference strength between *pf* = 2.4 and any larger value is negligible. Selection coefficients (*s*) were chosen in the range between 0.5 and 0.8 because lower and higher *s* values caused instability in population size, with one of the two homozygote phenotypes collapsing at a high rate (for further details on the influence of s values on phenotype coexistence and stability see Appendix S2.1).

As a control, 20 additional simulations were run separately with no recombination applied on offspring chromosomes (*s* = 0.5, *pf* = 0.3). Without recombination, the distance between ecological trait and preference loci has no influence on the association between the ecological trait and preference alleles, and therefore, there is no selection on the location of preference loci across the genome.

The average distance of preference loci from the ecological trait locus was calculated at the individual level, across both chromosomes, as was the average distance of neutral loci from the ecological trait locus, as a null model. The values were then averaged across all individuals in a single simulation and separately for individuals of AA and A′A′ phenotype. The average distances, at the population level, were recorded every generation, for the duration of 3000 generations. Values were then averaged across the 20 simulations for each combination of *s* and *pf*. The minimum average distance throughout the 3000 generations was taken as a measure of the strength of physical linkage between ecological traits and preference loci. The effect of *s* and *pf* values, as well as the interaction between them, on the minimum average distance between ecological trait and preference loci, was tested using Multiple linear regression.

#### 2.2.2 Observing preference loci locations across the population

To capture the locations of preference loci within the chromosomes across the population, the positions of all preference loci from a single simulation were recorded across all chromosomes in the population, separately for chromosomes with A and A′ alleles. An additional simulation was carried out without recombination, as a control. Both simulations were carried out with the lowest selection and mating preference strengths that were tested in the model (*s* = 0.5 and *pf* = 0.3), as a physical linkage between ecological trait and preference loci was strongest under these conditions. The positions were recorded after 1000 generations, when the average distance of preference loci from the ecological trait locus was found to be minimal across simulations. The number of chromosomes that held a preference locus at each location was counted across the population.

#### 2.2.3 Testing the association between physical linkage and level of assortative mating

To test whether there was a correlation between the strength of physical linkage between ecological trait and preference loci and the level of assortative mating, the average distance of preference loci from the ecological trait locus and the proportion of mating pairs that share the same phenotype were recorded from 30 simulations with the lowest selection coefficient and mating preference strength that were tested in the model (*s* = 0.5 and *pf* = 0.3). For comparison, the correlation was tested also for the average distance of neutral loci, which have no influence on mating preferences. The values were recorded after 700 generations, when variance in the proportion of phenotypically matching mating pairs across simulations was at a maximum, allowing to test whether differences among simulations in level of assortative mating are associated with differences in the strength of physical linkage.

Statistical analyses were performed using JMP Pro v.15.0.0 (2019) (SAS Institute, Inc.). All output data from simulations are provided in a public repository published in Dryad (https://doi.org/10.5061/dryad.q83bk3jm5; Schuldiner‐Harpaz et al., 2022).

## 3 RESULTS

**FIGURE 2:**
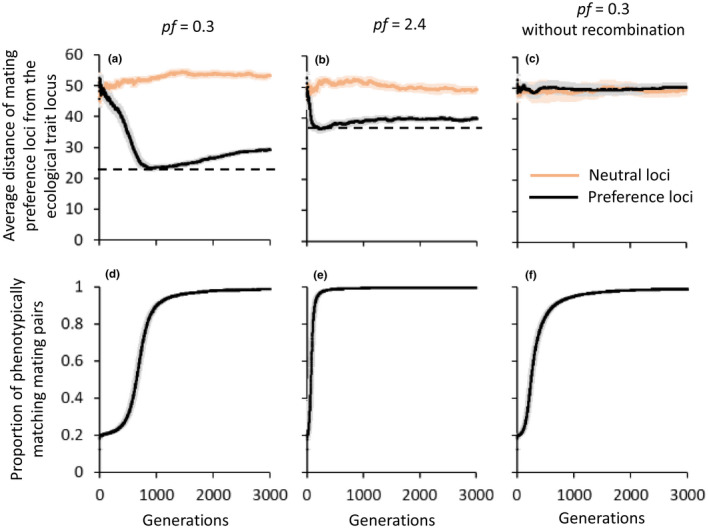
Changes in the level of physical linkage (a, b, c) and assortative mating (d, e, f) throughout 3000 simulated generations. Physical linkage is represented by the distance of mating preference loci (black lines) and neutral loci (orange lines) from the ecological trait locus. Results are presented for loci with alleles of preference for AA only, to prevent overlay, but are similar for loci with alleles of preference for A′A′. Horizontal dashed lines (a, b) mark the minimum average distance of preference loci from the ecological trait locus. Simulation were run with a selection coefficient of 0.5, and with low and high preference strength factors (*pf* = 0.3, a, d; and *pf* = 2.4, b, e, respectively), and low *pf* with no recombination applied on offspring chromosomes, as a control (c, f). For each of the three, average distances and proportions were calculated from 20 simulations. Light‐coloured shaded areas around the lines represent the standard error around the mean among simulations.

Strong and stable assortative mating developed in all simulations, together with a rapid decline in the abundance of the heterozygote, intermediate phenotype, as expected (Figure 2d–f; Appendix S4). Each of the homozygote phenotypes rapidly accumulated mating preference alleles for their own phenotype, followed by a decline in the rate of increase once assortative mating approached maximum (Appendix S4.2).

**FIGURE 3:**
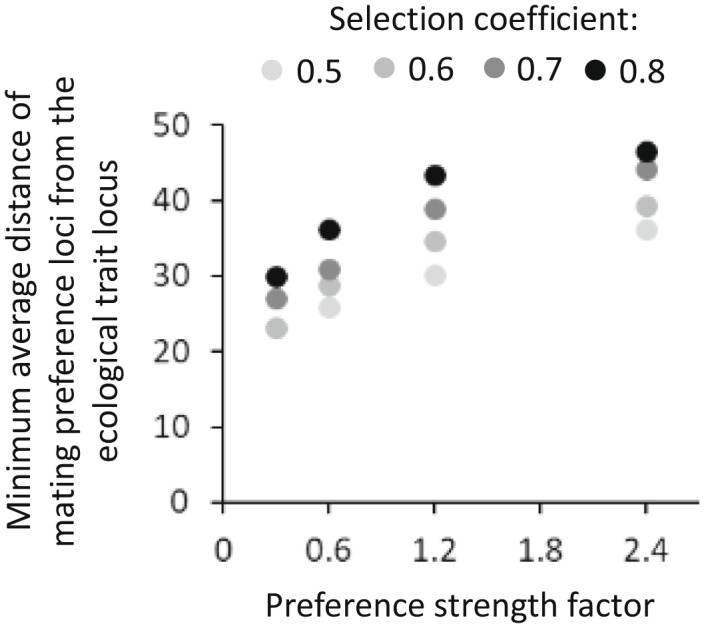
Negative influence of selection strength and mating preference strength on physical linkage. Physical linkage is represented by the minimum average distance of mating preference loci from the ecological trait locus, throughout 3000 simulated generations (marked with a dashed line in Figure 2a,b). Larger distances represent weaker physical linkage between the loci. Minimum average values are based on 20 simulations for each parameter combination. Results are presented for loci with alleles of preference for AA only, to prevent overlay, but are similar for loci with alleles of preference for A′A′.

Physical linkage, i.e., distance, between loci controlling mating preference and the locus controlling the ecological trait was significantly affected by the strength of both selection and mating preference (*F*_1,13_ = 47.5, *p* < 0.0001 and *F*_1,13_ = 105.7, *p* < 0.0001, respectively, for AA preference, and *F*_1,13_ = 24.2, *p* = 0.0003 and *F*_1,13_ = 52.9, *p* < 0.0001, respectively, for A′A′ preference). There was no significant interaction between selection and mating preference strength (*F*_1,12_ = 0.5, *p* = 0.5 and *F*_1,12_ = 0.02, *p* = 0.9, for AA preference and A′A′ preference, respectively). The minimum average distance between the ecological trait locus and preference loci throughout the simulation was smaller for weaker mating preference strengths (Figures 2a,b and 3) and for lower selection coefficients (Figure 3). However, we note that the negative correlation between selection strength and physical linkage is not always apparent when selection against heterozygotes is weaker compared to homozygotes in their unfavoured habitat (Appendix S3.5).

Simulations began with a rapid descent in the average distance of preference loci from the ecological trait locus (Figure 2a,b), which occurred in parallel to a rapid rise in assortative mating (Figure 2d,e). However, once the proportion of phenotypically matching mating pairs approached the maximum, the descent in average distance stopped. From this stage onwards, the average distance began to rise again at a very slow rate.

Assortative mating was established much more slowly for low values of the preference strength factor (*pf*) than for high values. For a selection coefficient of 0.5, 90% of mating pairs had matching phenotypes after 1005 generations when the preference was weak (*pf* = 0.3; Figure 2d), compared to only 136 generations when the preference was strong (*pf* = 2.4; Figure 2e). The more rapid rise in assortative mating when the mating preference was strong was associated with a quicker descent in the average distance between the ecological trait locus and preference loci (Figure 2b). However, the descent in average distance was also stopped at an earlier stage, which resulted in a higher minimum average distance (marked by a dashed line in Figure 2b).

The initial reduction in average distance between the ecological trait locus and preference loci did not occur for neutral loci with no influence on mating preference (grey lines in Figure 2a,b). Nor did it occur when no recombination was applied on offspring chromosomes, as a control for selection favouring physical linkage which cannot easily be broken by recombination (Figure 2c).

**FIGURE 4:**
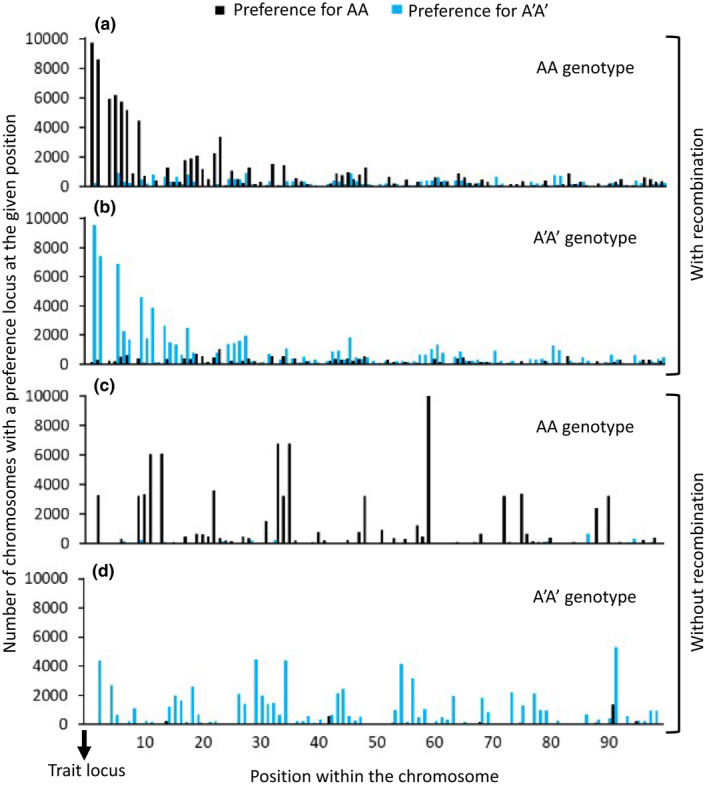
Frequency of preference loci positions across the population. Data are presented separately for loci with alleles of preference for AA (black bars) and loci with alleles of preference for A′A′ (blue bars). Data represent chromosomes of 10 000 individuals from a single example simulation with a selection coefficient of *s* = 0.5 and mating preference strength factor of *pf* = 0.3. The ecological trait locus is placed at the first position within the chromosome and therefore the position of mating preference loci also indicates their distance from the ecological trait locus. Loci positions are shown separately for a single simulation with (a, b) and without (c, d) recombination applied on offspring chromosomes. Positions were recorded after 1000 generations, when average distance of preference loci from the ecological trait locus is minimal across simulations.

The extent to which chromosomes with strong physical linkage between ecological trait and preference loci were favoured when mating preferences were weak was evident when examining the location of preference loci across chromosomes of all individuals in a single simulation. For the characteristic example simulation presented in Figure 4 (selection coefficient = 0.5, *pf* = 0.3), almost all chromosomes held a preference locus immediately adjacent to the ecological trait locus, and the majority held at least one additional preference locus within a distance of five loci from the ecological trait locus (Figure 4a,b).

In comparison, when no recombination was applied as a control, specific locations in the “genome” sequence held preference loci for a majority of individual chromosomes, but these were randomly dispersed at different distances from the ecological trait locus (Figure 4c,d). The average number of preference loci per chromosome in these example simulations was 10.87 (± 0.02).

**FIGURE 5:**
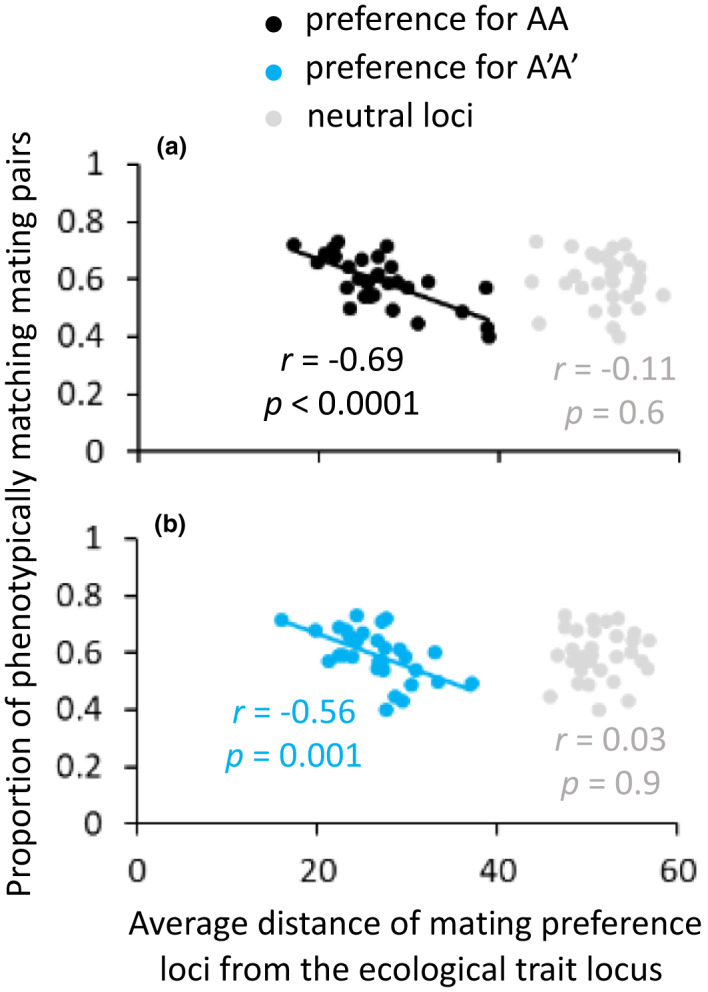
Negative correlation between distance of preference loci from the ecological trait locus and assortative mating. Data are based on 30 simulations with a selection coefficient of *s* = 0.5 and a mating preference strength factor of *pf* = 0.3, and are presented separately for loci with alleles of preference for AA (black dots), A′A′ (blue dots), and for neutral loci (grey dot), and separately for AA (a) and A′A′ (b) genotyped individuals. Values were recorded after 700 generations, when variance in the proportion of phenotypically matching mating pairs across simulations is at a maximum.

For a weak preference strength factor of 0.3 and selection coefficient of 0.5, the variance among simulations in the proportion of phenotypically matching mating pairs, as a measure of assortative mating, was highest after approximately 700 generations. At this stage, a significantly negative correlation was found between the average distance of mating preference loci from the ecological trait locus and the proportion of phenotypically matching mating pairs (*r* = −0.69, *df* = 28, *p* < 0.0001, and *r* = −0.56, *df* = 28, *p* = 0.001, for AA genotype and A′A′ genotype, respectively; Figure 5a,b). In other words, assortative mating was stronger in populations in which there was a stronger linkage between ecological trait and preference loci. No such correlation was found for neutral loci, with no influence on mating preference (*r* = −0.11, *df* = 28, *p* = 0.6, and *r* = 0.03, *df* = 28, *p* = 0.9, for AA genotype and A′A′ genotype, respectively; Figure 5a,b).

## 4 DISCUSSION

Our simulations suggest that the physical linkage between loci controlling ecological traits and mating preferences can evolve as a result of selection against intermediate, maladaptive hybrid phenotypes. Genomic architectures that maintained the association between ecological trait and preference alleles were favoured when there was a high risk of the association breaking down. However, our model also showed that although a tight physical linkage between ecological trait and preference loci can facilitate the evolution of assortative mating, it is not essential. These findings reflect the genetic architecture of pre‐mating isolation found in real systems. Studies on a range of organisms provide evidence of both strong physical linkage between loci controlling ecological traits and mating preferences (Bay et al., 2017; Hawthorne and Via, 2001; Merrill et al., 2019) and strong linkage disequilibrium despite no physical linkage (Hench et al., 2019).

We demonstrated that a strong physical linkage between ecological trait and preference loci evolves most readily if mating preferences are initially weak. Genomes with strong physical linkage were favoured only when the additional influence of each individual preference allele was small. This meant assortative mating developed gradually, allowing for separate phenotypes to mate more frequently and for recombination to shuffle phenotypes and preferences such that an individual might prefer to mate with others that do not match its own phenotype. Under such conditions, there is strong selection pressure favouring genomes with the strong physical linkage between ecological trait and preference loci, that is less likely to be disassociated by recombination. In this sense, the forces that drive physical linkage in our model resemble those that drive pre‐zygotic isolation during reinforcement. In both cases, once assortative mating is the strong selection no longer favours elements that promote reproductive isolation.

Our demonstration that physical linkage evolves only where preference strength is weak is further strengthened by the fact that physical linkage in our simulations slowly weakened once assortative mating was established. The weakening of physical linkage indicates that random processes controlled the location of preference loci once physical linkage to the ecological trait locus was no longer essential. New preference alleles that arose after the establishment of assortative mating would become and remain in linkage disequilibrium with the trait allele regardless of their location simply because gene flow between the two homozygote populations was already suppressed. This long‐term outcome is revealed due to our modelling approach, in which preference is controlled by multiple loci that are added over time. Such a pattern would not have been revealed if linkage strength was controlled by modifiers of recombination rate, as modelled in previous theoretical studies (Charlesworth and Charlesworth, 1979; Pylkov et al., 1998; Trickett and Butlin, 1994), rather than by the physical location of loci. The fading of physical linkage over time in our model suggests that the absence of physical linkage in current genomes could also be a result of previously existing linkage that was lost over time due to random processes, once speciation was complete. In reality, the probability of losing linkage in this way would depend on the likelihood that loci controlling assortative mating mutate back to an ancestral state.

Selection strength was found to have an overall negative effect on physical linkage between ecological trait and preference loci. Selection against intermediate, hybrid, phenotypes may influence physical linkage in two opposite manners. On the one hand, it drives assortative mating and therefore also physical linkage, which facilitates assortative mating. On the other hand strong assortative mating alleviates the force driving physical linkage, as mentioned above. Therefore, the strength of selection has both a positive direct and negative indirect influence on physical linkage. The overall negative association found between selection strength and physical linkage indicates that the latter has a higher impact than the former. Similarly, in a theoretical study of genomic architectures of adaptation, Yeaman and Whitlock (2011) found that under strong selection, tight clustering of locally adapted alleles is not as advantageous as when selection on the alleles is weak.

Our study joins earlier theoretical studies in a slightly different context, which have shown that linkage disequilibrium can develop between alleles that contribute to local adaptation, even without physical linkage between the relevant loci (Feder et al., 2012; Flaxman et al., 2014; Yeaman and Whitlock, 2011). However, several models have also shown that clustering of divergent loci can evolve in biologically realistic time scales (Yeaman, 2013; Yeaman and Whitlock, 2011), and that physical linkage between divergent alleles plays a more prominent role in speciation when new divergent alleles are of small effect (Feder et al., 2012; Yeaman and Whitlock 2011). These findings are in accordance with conclusions from our own model, in which tight physical linkage was found when the effect size of preference loci was small (low *pf* values). If loci of small effect on mating preferences tend to accumulate closer to ecological trait loci compared to loci of large effect, physical linkage would be harder to detect empirically, due to large effect loci overshadowing the effect of small effect loci.

Our model also shows that physical linkage facilitates assortative mating. Populations in which physical linkage between ecological trait and preference loci was stronger showed higher levels of assortative mating, during the phase at which assortative mating was not yet complete. Consequently, there is a complex balance of forces acting during speciation. The physical linkage between ecological trait and preference loci facilitates assortative mating, by strengthening the association between matching ecological trait and preference alleles. In turn, strong assortative mating prevents further development of physical linkage because it reduces the probability of hybridization and therefore removes the force favouring strong physical linkage. Therefore, it seems physical linkage both influences and is governed by assortative mating in a negative feedback mechanism. A similar argument was made regarding reinforcement, which “pulls the rug out from under itself” (Coyne and Orr, 2004). In reinforcement, the frequency of hybridization determines the strength of selection for pre‐zygotic isolation. Therefore, reinforcement drives pre‐zygotic isolation and at the same time is weakened as pre‐zygotic isolation increases (Moore, 1957; Spencer et al., 1986).

We note that our model represents a system in which speciation is highly likely, because the trait under divergent selection serves also as a mating cue (a ‘magic trait’). Therefore, once mating preference alleles are added, the build up of an association between divergent selection and assortative mating is inevitable. This is in contrast to Felsenstein's model (1981), in which speciation requires the build up of an association between alleles at an independent locus that causes assortative mating and at a pair of epistatically interacting loci that cause disruptive selection. In the latter, linkage disequilibrium between divergent alleles is unlikely to be maintained without reduced recombination. Whereas in our model, the association between ecological trait alleles and preference alleles can develop despite recombination, as long as selection and mating preferences are strong enough.

Despite the high likelihood of speciation, our model represents a challenging starting point in which gene flow among populations from separate habitats is at a maximum. Populations from the two habitats merge into a single mating pool, with an equal probability to encounter all phenotypes when choosing a mate. It is interesting to consider the influence that limiting the contact among the two populations would have on the evolution of physical linkage between ecological trait and preference loci. Theoretical studies on “genomic island of divergence”, suggest that clustering between locally adapted alleles should decrease with decreasing migration rates (Kirkpatrick and Barton, 2006; Yeaman and Whitlock, 2011). We assume that decreasing the level of mixture among populations from the two habitats in our model would have a similar effect on physical linkage between ecological trait and preference loci. At low‐migration rates, separate phenotypes are less likely to hybridize, and therefore physically linked ecological trait, and preference loci have less advantage over non‐linked loci.

There is still much that remains unknown about changes in genomic architecture during speciation. Identifying the evolutionary forces that shape the structure of diverging genomes is critical for advancing our understanding of speciation. Yet, most theoretical studies on the evolution of linkage disequilibrium between divergent alleles consider only the influence of physical linkage on speciation, rather than studying the evolution of physical linkage itself. Here, we identified factors that shape the location of mating preference loci relative to ecological trait loci. Subsequently, we are able to suggest under which conditions physical linkage is essential for assortative mating to evolve and under which conditions it is unnecessary. Empirical evidence of the physical linkage between loci controlling ecological traits and mating preferences is still scarce. Therefore, additional study systems are needed to verify our predictions regarding the association between mating preference strength and physical linkage between loci controlling ecological and mating triats.

## AUTHOR CONTRIBUTIONS

T. Schuldiner‐Harpaz, R. M. Merrill and C. D. Jiggins conceived and designed the study, T. Schuldiner‐Harpaz developed and analysed the models, T. Schuldiner‐Harpaz, R. M. Merrill and C. D. Jiggins prepared the manuscript.

## CONFLICT OF INTEREST

The authors have no conflict of interest to declare.

### PEER REVIEW

The peer review history for this article is available at https://publons.com/publon/10.1111/jeb.14105.

## Supporting information


Appendix S1

Appendix S2

Appendix S3

Appendix S4

## Data Availability

Model files, including source code, are openly available at the NetLogo User community website, via the following links: http://ccl.northwestern.edu/netlogo/models/community/physical_linkage_one_trait_locus; http://ccl.northwestern.edu/netlogo/models/community/physical_linkage_two_trait_loci; The simulations output data that was used for analyses is openly available via the following link: https://doi.org/10.5061/dryad.q83bk3jm5.
