# Supplementary material for: Evolution of physical linkage between loci controlling ecological traits and mating preferences
Source: J Evol Biol. 2022 Oct 5;35(11):1537–47. doi: 10.1111/jeb.14105 (PMC9827829; doi:10.1111/jeb.14105)
Supplement: Supplementary file 1 — Appendix S1 [file JEB-35-1537-s002.docx]

**Appendix S1. Model Overview, Design concepts and Details (ODD)**

Complete model description following the ODD (Overview, Design concepts and Details) protocol for individual-based models (Grimm et al., 2006, 2010):

1. **Purpose**

The model was designed to explore evolutionary changes in the relative location of loci contributing to reproductive isolation due to selection pressure against intermediate phenotypes. The model is inspired by the colour patterns and mating behaviours of *Heliconius* butterflies but applies for a wide range of biological systems.

1. **Entities, state variables, and scales**

The model consists of 10000 individuals with an even male:female ratio. Individuals are diploid with a single pair of homologous chromosomes, each containing a sequence of 100 loci, defined together as the “genome”. The first locus controls an ecological trait, for which there are two possible alleles, with an initially equal frequency in the population: A or A’. The alleles are codominant and therefore the three possible genotypes produce three separate phenotypes: AA, A’A’ homozygotes or an intermediate, heterozygote AA’ phenotype. The ecological trait is subject to selection, but also serves as a mating cue and can therefore be considered a “magic trait” (Servedio et al. 2011).

Mutations that cause a preference to mate with either AA or A’A’ phenotypes (preference loci), as well as neutral mutations for comparison, occur at a single randomly chosen locus in one percent of individuals every generation. The higher the number of preference loci for AA in the genome, compared to the number of A’A’ preference loci, the higher the probability to mate with an individual of AA phenotype (see Submodels section for detailed description).

The modelled environment comprises two habitats, such that selection favours AA genotype in one habitat and A’A’ genotype in the other. The habitats are equally maladaptive for the heterozygote AA’ phenotype and for the unfavoured homozygote phenotype in each habitat. The adaptive allele is thus effectively recessive within each habitat, even though there are three distinct phenotypes AA, AA’ and A’A' (that play a role in mate choice, see below). Selection is modelled to reflect a scenario of two separate phenotypic optima, with sub-optimal hybrid phenotypes. Habitats are represented in the model in a non-spatial manner. Individuals remain in the habitat to which they are initially assigned, except while mating.

Time steps in the model correspond to discrete, non-overlapping generations. Each simulation was run for 3000 generations.

1. **Process overview and scheduling**

Each generation, several stages are executed in the following order: (1) formation of mating pairs, (2) reproduction, (3) recombination of offspring chromosomes and addition of randomly placed neutral mutations or mutations causing mating preference, (5) ecological selection, and (6) density dependent regulation of population size. For details on each process see ‘Submodels’ section.

1. **Design concepts**

*Basic principles.*

The model design is based on several Basic principles: (i) selection favours distinct phenotypes of the ecological trait and acts against intermediate phenotypes that arise when separate phenotypes mate, thereby promoting assortative mating based on the trait phenotype. Therefore, the ecological trait is regarded as a ‘magic trait’, which is subject to divergent ecological selection and also contributes to non-random mating (Servedio et al., 2011). (ii) Genetic elements that control the ecological trait evolve first, followed by the evolution of genetic elements that control mating preferences, as is assumed to have occurred in *Heliconius* (Jiggins et al., 2004). The latter can potentially evolve at various places within the genome, and within various distances from ecological trait loci. (iii) Recombination has the potential to break up associations between specific ecological trait and preference alleles (Felsenstein, 1981). Therefore, the physical distance between ecological trait and preference alleles within the genome plays an important role in the development of assortative mating.

*Emergence.*

Given the disadvantage of offspring with an intermediate phenotype, assortative mating is expected to emerge. Mutations that cause mating preference for the AA or A’A’ genotypes are expected to accumulate on chromosomes that carry the A or A’ allele, respectively. However, it is not straightforward under which conditions selection will favour genomes in which preference loci accumulate nearby the ecological trait locus.

*Stochasticity*.

The model includes several procedures that are determined stochastically, to represent random events that take place in reality. These include matching of individuals with potential mating partners, choice of two of the four parent chromosomes which will be passed on to each of their offspring separately, the cross-over point for recombination, individual genomes that undergo mutation (either neutral or contributing to mating preference), and the location of each mutation within the genome.

Alongside these central stochastic procedures, there are several components of the model that are determined stochastically to maintain events or behaviours at a specified frequency. These include the initial distribution of individuals among the two habitats, determining the ecological trait locus alleles of initial individuals, the decision whether to mate with a potential mating partner, and death due to selection and due to density-dependent population size regulation.

*Observation.*

The average distance of each type of preference loci and of neutral mutations from the ecological trait locus is recorded for each individual across both chromosomes. The values are then averaged across all individuals, separately for individuals of AA and A’A’ phenotype, at every generation.

The proportion of phenotypically matching mating pairs, in which both male and female are of AA phenotype or of A’A’ phenotype, is recorded every generation, as a measure of assortative mating.

1. **Initialization**

At the beginning of each simulation, 10000 individuals, half male and half female, are randomly assigned to one of the two habitats. The alleles at the ecological trait locus on each of the two chromosomes of each individual are randomly chosen at an equal probability for the A and A’ alleles. Neutral mutations or ones that cause mating preferences are then added to a single randomly chosen locus in one percent of the population.

1. **Input data**

The model does not use input data to represent time-varying processes.

1. **Submodels**
2. *Formation of mating pairs*

The mating choice is made by the females, who are randomly paired with males from both habitats, reflecting a scenario of unrestricted movement of males between habitats when searching for a mate. A female will mate with a male with which she is paired at a probability that depends on the strength of her preference for his phenotype (see below). If a female decides not to mate, she is paired sequentially with a maximum of ten random males until she mates. If she does not mate with the tenth male with which she is paired, she is taken out of the mating pool and does not reproduce. Limiting the number of rejections to ten avoids simulations from running endlessly, while maintaining negligible costs of female mating preference (Schneider and Bürger, 2006; Kopp and Hermisson, 2008). See supplementary material (S2.4) for details on the sensitivity of model results to changes in the maximum number of males with which a female is paired.

The probability of a female to mate with a male with which she is paired is described by the following equations (Fig. 1):

$$P_{A}=\frac{1}{1+e^{-d\cdot pf}}$$

$$P_{A'}=1-P_{A}$$

Where *P_A_* and *P_A’_* are the probabilities to mate with a male of phenotype AA and A’A’, respectively. *d* is the difference between the number of AA preference loci and the number of A’A’ preference loci in the female’s genome. The higher the number of AA preference loci, compared to A’A’ preference loci, the larger the probability to mate with an AA phenotyped male, and vice versa. A preference strength factor (pf) determines how strong the contribution of each additional preference locus is.

The probability to mate with a male of AA’ phenotype is constant for all individuals and equals 0.5. This represents a case in which hybrids, represented by the heterozygotes in this model, exhibit intermediate phenotypes which are partially attractive to individuals who prefer to mate with one of the distinct phenotypes, represented as homozygotes in this model. Robustness of model results to changes in this basic assumption are detailed in the supplementary material (S3.1).

The preference strength factor (*pf*) is used to control the rate at which strong mating preferences accumulate across the population. Varying the value of *pf* allows testing the influence of mating preference strength on the development of physical linkage between ecological trait and preference loci.

Sexual selection only exists in our model if divergent selection is not strong enough to maintain stable coexistence of the two homozygote phenotypes. Under such a scenario, slight differences in the frequencies of the homozygote phenotypes will cause a disadvantage to the less frequent phenotype in finding a mating partner, resulting in a continued decline in frequency and rapid collapse of the less frequent phenotype. (see supplement S2.1 for further detail).

1. *Reproduction*

Only individuals who have found a mating partner in the previous procedure will reproduce. Each mating pair produces four offspring, two males and two females. The number of offspring was chosen to keep the size of the new generation above carrying capacity, before selection and density dependent regulation take place, to avoid population collapse.

Each offspring receives one paternal and one maternal chromosome, randomly chosen from the two chromosomes of each parent. Offspring are assigned the same habitat as their mother, to ensure that the mating choice has a direct influence on the survival of offspring and is therefore subject to natural selection. Following reproduction, the parent generation dies.

1. *Offspring recombination and mutation*

Each offspring genome undergoes recombination between the two homologous chromosomes. Recombination occurs at one, randomly chosen, cross-over point within the chromosomes. The content of the “genome” sequence after the cross-over point is exchanged between the two homologous chromosomes.

Recombination can break apart linkage disequilibrium between ecological trait and mating preference alleles, both in the model and in reality. It is therefore the force that drives physical linkage between loci controlling the ecological trait and loci controlling mating preferences. The recombination does not change the offspring genotype or number of preference alleles, and therefore has no influence on the offspring itself, and can be seen as representing recombination in the offspring gametes, as preparation for mating.

After recombination is completed, one percent of individuals receive a mutation in a single locus, causing mating preference for either AA or A’A’ phenotypes (preference loci), or having no influence on mating preferences (neutral loci), for reference. The three possible mutations occur at equal probabilities, at a randomly chosen location within the “genome”, excluding the first locus, which controls the ecological trait. A given locus can carry only one mutation. Therefore, changes in preference loci are possible, as are back mutations, if a neutral mutation occurs at a locus that was previously a preference locus. Because mutations occurs at a single locus, chosen from two chromosomes, in one percent of individuals, the mutation rate per locus is $5\cdot{10}^{-5}$. See supplementary material (S2.3) for details on the sensitivity of model results to changes in the percent of individuals that undergo mutations every generation.

We chose to add randomly placed mutations that cause mating preferences based on the assumption that physical linkage is formed by co-option of genes that are already within physical linkage with the ecological trait locus, rather than by transposition of genetic elements that control preference from other regions in the genome. In *Heliconius*, for example, there is no evidence of transposition around colour pattern genes, or chromosomal inversions that might be involved in maintaining species barriers among *Heliconius melpomene* and *H. cydno* (The Heliconius Genome Consortium, 2012; Davey et al., 2017).

1. *Ecological selection*

Selection favours AA phenotype in one habitat and A’A’ phenotype in the second habitat. Phenotypes that are not favoured in each of the habitats are subject to selection. Selection is modelled to reflect a scenario of two separate phenotypic optima, with sub-optimal hybrid phenotypes. The strength of selection is fixed per simulation and controlled by a selection coefficient (*s*), which determines the proportion of maladapted individuals that will die due to selection. Robustness of model results to changes in the relative selection level against heterozygote, intermediate, phenotypes are detailed in the supplementary material (S3.2).

1. *Density dependent regulation*

Each of the two habitats has a carrying capacity of 5000 individuals. The probability of each individual to die due to density dependent regulation equals the number of excess individuals in the respective habitat divided by the total number of individuals in that habitat. Separate density dependent regulation of population size in each habitat is based on the assumption that individuals depend on separate, limited, resources in each habitat, making the model a soft selection model (Wallace, 1975).

The sensitivity of model results to changes in the values of parameters other than *s* and *pf* (i.e. maximum attempts to find a mate, mutation rate, genome length and carrying capacity), as well as the robustness of results in response to changes in basic model assumptions, are described in the supplementary material (S2 and S3, respectively).
