# Supplementary material for: Evolution of physical linkage between loci controlling ecological traits and mating preferences
Source: J Evol Biol. 2022 Oct 5;35(11):1537–47. doi: 10.1111/jeb.14105 (PMC9827829; doi:10.1111/jeb.14105)
Supplement: Supplementary file 2 — Appendix S2 [file JEB-35-1537-s003.docx]

**Appendix S2. Sensitivity of the model outcomes to changes in parameter values.**

The following table details all model parameters, the values that were used for each parameter in the model and the wider range values that were tested to examine the sensitivity of model outcomes to changes in each parameter. Details of the test results are outlined below the table.

| **Parameter** | **Description** | **Values used in the model** | **Values tested for model sensitivity** | **Sensitivity analysis outcome** |
| --- | --- | --- | --- | --- |
| 1. Selection coefficient (*s*) | The proportion of maladaptive individuals that die each generation as a result of selection | 0.5, 0.6, 0.7, 0.8 | 0, 0.1, 0.2, 0.3, 0.4, 0.9, 1 | Low and high selection coefficient values (*s* < 0.5 and *s* > 0.9) caused the frequent collapse of one of the homozygote phenotypes. |
| 1. Preference strength factor (*pf*) | A factor that determines the addition of each preference allele to overall mating preference strength | 0.3, 0.6, 1.2, 2.4 | NA | NA |
| 1. Mutation rate | The proportion of individuals that undergo a single mutation at a randomly chosen locus every generation | 0.01 | 0.001, 0.005, 0.01, 0.05, 0.1, 1 | Reducing the mutation rate did not change model conclusions regarding stronger physical linkage for weaker mating preference strengths and weaker selection. |
| 1. Maximum attempts to find a mate | The maximum number of potential male mating partners with which a female is paired sequentially | 10 | 2, 4, 6, 8, 10, 12, 14, 16 | A low number of maximum attempts (four or less) resulted in the collapse of one of the homozygote phenotypes. Decreasing the number of attempts to six had no influence on the overall outcome of the model. Increasing the maximum number of attempts above 10 had no influence on the proportion of individuals that do not mate. |
| 1. Genome length | The number of loci in the genome sequence | 100 | 200 | Increasing genome size had an influence only on the scale of distance between loci, but not on the general trends. |
| 1. Carrying capacity | The number of individuals in the simulated environment | 10000 | 1000, 5000 | Reducing population size had an influence on population stability and on the co-existence of the two homozygote phenotypes, but had no influence on the general trends of physical linkage. |

1. **Selection coefficient (*s*)**

Selection acts as a stabilizing force in the model, by maintaining similar frequencies of the two homozygote phenotypes. If one of the homozygote phenotypes becomes, by chance, slightly less frequent than the other, it will have a disadvantage in finding a mating partner that matches its phenotype, resulting in a continued decline in frequency and rapid collapse of the less frequent phenotype. Selection acts as a stabilizing force by maintaining the advantage of the less frequent phenotype in the habitat in which it is favoured and preventing its displacement by the second phenotype. Therefore, selection that is too weak (*s* < 0.5) is not sufficient to maintain the advantage for each phenotype in the habitat in which it is favoured, resulting in the frequent collapse of one phenotype, because it is displaced by the other in both habitats. On the other hand, selection that is too strong (*s* > 0.8) generates frequent changes in population size and relative phenotype frequencies, leading to the decline of the less frequent phenotypes as described above.

In accordance, one of the two homozygote phenotypes collapsed in the majority of simulations in which the selection coefficient was low (*s* = 0, 0.1, 0.2, 0.3, 0.4) or high (*s* = 0.9, 1). The proportion of unstable simulations for each low and high selection coefficient and low and high preference strength factor (*pf*), are detailed in the following table:

|  | *pf* = 0.3 | *pf* = 2.4 |
| --- | --- | --- |
| *s* = 0 | 19/20 | 20/20 |
| *s* = 0.1 | 20/20 | 20/20 |
| *s* = 0.2 | 20/20 | 20/20 |
| *s* = 0.3 | 20/20 | 17/20 |
| *s* = 0.4 | 20/20 | 0/20 |
| *s* = 0.9 | 20/20 | 18/20 |
| *s* = 1 | 20/20 | 20/20 |

1. **Preference strength factor (*pf*)**

The *pf* is used to control the rate at which strong mating preferences accumulate across the population. The maximum value was set to 2.4 because for this value an excess of only one preference allele for a specific phenotype causes individuals to mate with the preferred phenotypes at a probability greater than 0.9 (Fig. 1). Therefore, there was no need to test the model outcome for higher *pf* values, because the difference in overall preference strength for higher *pf* values would be negligible.

1. **Mutation rate**

Every generation, one percent of individuals undergo a single mutation at a random locus. To test the sensitivity of model results to changes in the proportion of individuals that undergo mutation (mutation rate), we examined two additional lower mutation rates (0.001 and 0.005) and three higher rates (0.05, 0.1, 1). The simulations were carried out separately for the lowest and highest preference strength factors and selection coefficients which were tested in the model (*pf* = 0.3 and *pf* = 2.4, and *s* = 0.5 and *s* = 0.8, respectively). However, for the lowest mutation rate and low values of *pf* and *s*, at least one of the phenotypes collapsed in five of the simulations. This could be due to the very low number of individuals with specific mating preferences, resulting in frequent mating among phenotypes and low survival rate of offspring.

In line with the main conclusions from the model, physical linkage between ecological trait and preference loci was stronger for weaker mating preferences and weaker selection for all mutations rates, apart from a mutation rate of 1, for which assortative mating developed too quickly for physical linkage to evolve. Higher mutation rates resulted in weaker physical linkage, due to faster establishment of assortative mating. Lowering the mutation rate below 0.01 increased the variance among individuals in distance of preference loci from the ecological trait locus and did not necessarily result in substantially stronger physical linkage (Fig. S2.3).


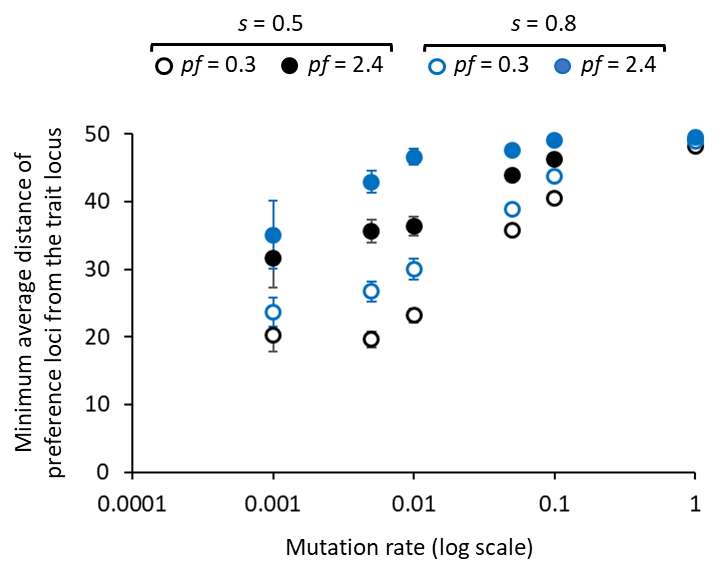


**Fig. S2.3.** Minimal average distance of preference loci from the ecological trait locus for a range of mutation rates, selection coefficients of *s* = 0.5 (black markers) and *s* = 0.8 (blue markers), and preference strength factors of *pf* = 0.3 (unfilled markers) and *pf* = 2.4 (filled markers). Larger distances represent weaker physical linkage between the loci. Results are presented for loci with alleles of preference for AA only, to prevent overlay, but are similar for loci with alleles of preference for A’A’. Minimum average values are based on 10 simulations for each parameter combination.

1. **Maximum attempts to find a mate**

Each female is paired with a random male and will mate with him at a probability that depends on the strength of her preference for his phenotype. If a female decides not to mate, she is paired sequentially with a maximum number of random males until she mates. If she does not mate with the last male with which he is paired, she does not reproduce.

Limiting the number of males with which a female is paired is done to avoid simulations from running endlessly. However, the maximum number must be large enough to keep the percentage of unmated females low. To determine the optimal maximum number of attempts, we tested various maximum values ranging from two to 16, in increments of two. For a maximum of two and four attempts, the number of individuals that found a mate successfully was relatively low, causing one of the two homozygote phenotypes to collapse after a small number of generations.

The average and maximum percentages of unmated females reach stable, low, values of approximately 0.8% and 4-5%, respectively, at a little less than a maximum of ten attempts. Increasing the maximum number of attempts beyond ten does not decrease the percentage of unmated females (Fig. S2.4.1). Therefore, ten was the value chosen for the modelled parameter.


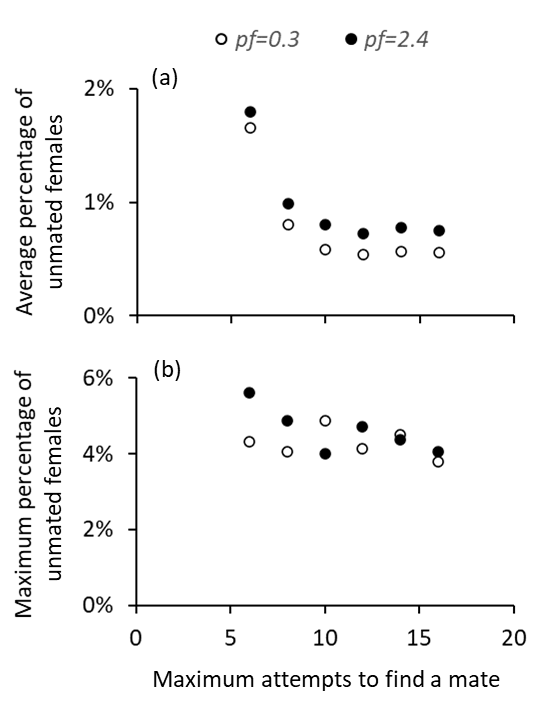


**Fig. S2.4.1.** The influence of the value of maximum attempts to find a mate on the average (a) and maximum (b) percentage of females that remain unmated across 3000 generations of a single simulation. Simulations were run with a selection coefficient of *s* = 0.5 and separately for low and high preference strength factors (*pf* = 0.3 and *pf* = 2.4, respectively).

To test the sensitivity of the model outcome to a lower value of maximum attempts, simulations were carried out for a maximum of six attempts to find a mate, as further reduction in the number of attempts would cause population instability as mentioned above. The simulations were carried out separately for the lowest and highest preference strength factors and selection coefficients which were tested in the model (*pf* = 0.3 and *pf* = 2.4, and *s* = 0.5 and *s* = 0.8, respectively). Reducing the maximum number of attempts to six had no influence on the main conclusions from the model, i.e. physical linkage between ecological trait and preference loci remained stronger for weaker mating preferences and weaker selection (Fig. S2.4.2).


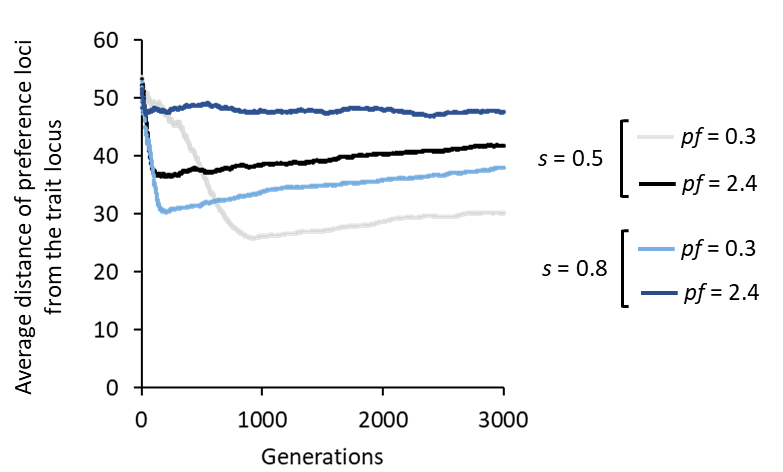


**Fig. S2.4.2.** Changes in the level of physical linkage throughout 3000 simulated generations, with a selection coefficient of *s*=0.5 (black and grey lines) or *s*=0.8 (dark and light blue lines), and with low and high preference strength factors (*pf*=0.3, grey and light blue lines; and *pf*=2.4, black and dark blue lines, respectively). Physical linkage is represented by the distance of mating preference loci from the trait locus. Results are presented for loci with alleles of preference for AA only, to prevent overlay, but are similar for loci with alleles of preference for A’A’. For each of the four combinations average distances were calculated from 10 simulations.

1. **Genome length**

To test whether the model outcome may be sensitive to the number of loci in individual chromosomes (genome length), additional simulations were carried out with a larger genome of 200 loci, compared to the 100 loci long genome used in the original simulations. The simulations were carried out with a selection coefficient of 0.5 and low and high preference strength factors (*pf* = 0.3 and *pf* = 2.4). Genome length influenced only the scale of distance between preference loci and the ecological trait locus but had no influence on the general trend in results (Fig. S2.5).


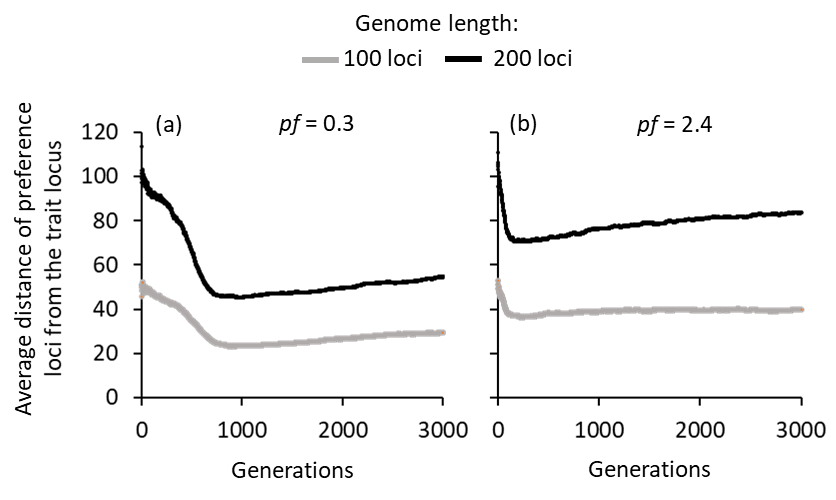


**Fig S2.5.** Changes in the level of physical linkage throughout 3000 simulated generations, for 100 and 200 loci long genomes (grey and black lines, respectively). Simulation were run with a selection coefficient of 0.5 and preference strength factors of *s* = 0.3 (a) and *s* = 2.4 (b). Physical linkage is represented by the distance of mating preference loci from the trait locus. Results are presented for loci with alleles of preference for AA only, to prevent overlay, but are similar for loci with alleles of preference for A’A’. For each combination of genome length and preference strength factor average distances were calculated from 10 simulations.

1. **Population size**

To test the sensitivity of the main model outcomes to a reduction in population size we carried out simulations with a population size of 1000 and 5000 individuals and compared the simulation outcome to the original population size of 10000 individuals. Each was carried out separately for the lowest and highest preference strength factors and selection coefficients which were tested in the model (*pf* = 0.3 and *pf* = 2.4, and *s* = 0.5 and *s* = 0.8, respectively). The decrease in population size reduced population stability and resulted in the frequent collapse of one of the two homozygote phenotypes, as detailed in the following table:

| Selection coefficient (*s*) | Preference strength factor (*pf*) | Proportion of simulations in which one of the two homozygote phenotypes collapsed | |
| --- | --- | --- | --- |
|  |  | 1000 individuals | 5000 individuals |
| 0.5 | 0.3 | 9/20 | 0/20 |
|  | 2.4 | 0/20 | 0/20 |
| 0.8 | 0.3 | 12/20 | 5/20 |
|  | 2.4 | 11/20 | 2/20 |

For simulations in which the population remained stable, population size had no influence on the main conclusions from the model. Physical linkage between ecological trait and preference loci remained stronger for weaker mating preferences and weaker selection (Fig. S2.6). However, we note that physical linkage was substantially weakened for a population size of 1000, when selection was strong (*s* = 0.8) and mating preferences were weak (*pf* = 0.3). This was also the combination for which the highest number of unstable simulations was recorded, which may explain the decrease in physical linkage.


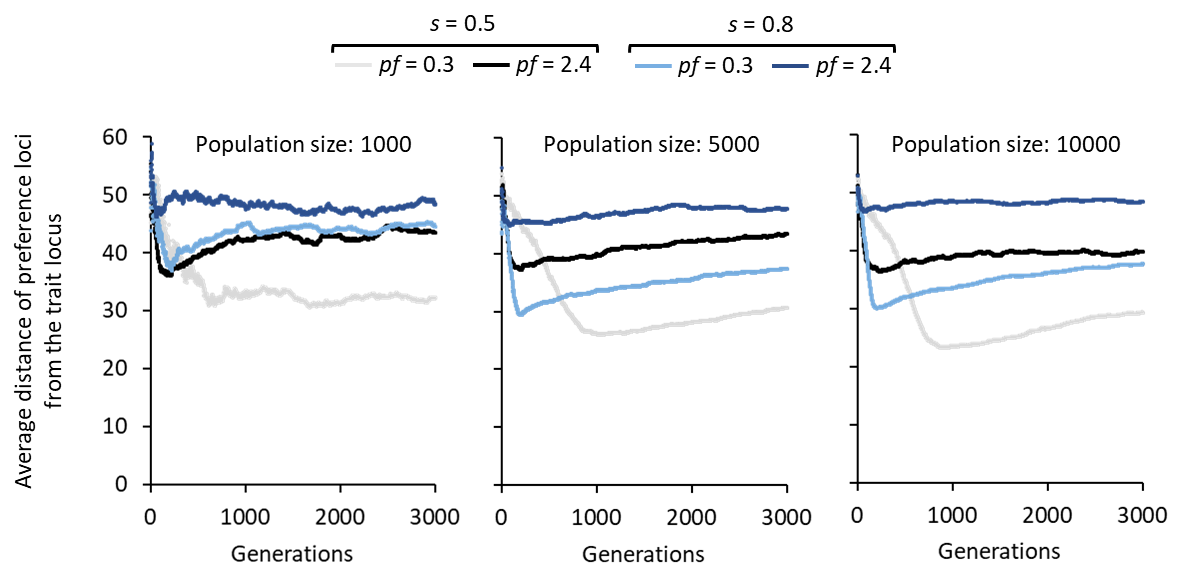


**Fig. S2.6.** Changes in the level of physical linkage throughout 3000 simulated generations, for three different population sizes. Simulations were run with a selection coefficient of *s*=0.5 (black and grey lines) or *s*=0.8 (dark and light blue lines), and with low and high preference strength factors (*pf*=0.3, grey and light blue lines; and *pf*=2.4, black and dark blue lines, respectively). Physical linkage is represented by the distance of mating preference loci from the trait locus. Results are presented for loci with alleles of preference for AA only, to prevent overlay, but are similar for loci with alleles of preference for A’A’. For each parameter combination average distances were calculated from 10 simulations.
