# Supplementary material for: Evolution of physical linkage between loci controlling ecological traits and mating preferences
Source: J Evol Biol. 2022 Oct 5;35(11):1537–47. doi: 10.1111/jeb.14105 (PMC9827829; doi:10.1111/jeb.14105)
Supplement: Supplementary file 3 — Appendix S3 [file JEB-35-1537-s004.docx]

**Appendix S3. Robustness of model outcomes in response to changes in basic assumptions**

Several assumptions were made while formulating the model, some of which are not central to the research questions highlighted in this study. The robustness of the main model outcomes was tested in response to changes in these assumptions. For each of the assumptions listed below we tested the influence of alternative assumptions on the main trends, i.e. does physical linkage between loci controlling the ecological trait and mating preferences remain stronger when mating preferences and selection pressure are weaker.

The following table lists the alternative assumptions that were tested for each original assumption. Further details of each assumption and simulation results are outlined below the table.

|  | **Original assumption** | **Alternative assumptions** |
| --- | --- | --- |
| 1. | Males are matched with a single female and mate only once. | Males may mate with multiple females. |
| 2. | Preference to mate with a heterozygote is intermediate between the preference for each of the homozygote phenotypes. | Preference to mate with a homozygote causes (a) stronger, or (b) weaker preference to mate with a heterozygote. |
| 3. | Recombination occurs once in each parent and offspring inherit one of two possible haplotypes. | Recombination occurs separately for each gamete, producing multiple possible haplotypes across offspring.  Two recombination rates were tested: recombination occurs at a probability of either 1 or o.5 per chromosome. |
| 4. | Mutation rate is implemented at the population level: mutations occur at a single locus in 1% of the population. | Mutation rate is implemented at the single locus level: each locus has a probability of $5\cdot{10}^{-5}$ to undergo mutation. |
| 5. | Selection acts equally against all unfavourable phenotypes in each habitat. | The heterozygote, intermediate, phenotype shares some of the favourable characters of each homozygote phenotype, causing it to be partially favoured in each habitat. |
| 6. | The ecological trait is controlled by a single locus. | The ecological trait is controlled by two loci. |

**S3.1 Number of mating partners per male**

In the original model, males were allowed to mate only once, with a single female. Here we tested whether allowing males to mate with multiple females would influence (i) the main trends in simulation results, and (ii) population stability, i.e. whether at very low and very high selection pressure one of the homozygote phenotypes collapses, as occurred frequently in the original model version.

Allowing males to mate with multiple females did not influence the general trends in simulation results. Physical linkage between trait and preference loci remained stronger for weaker preference strength and weaker selection pressure (Fig. S3.1).


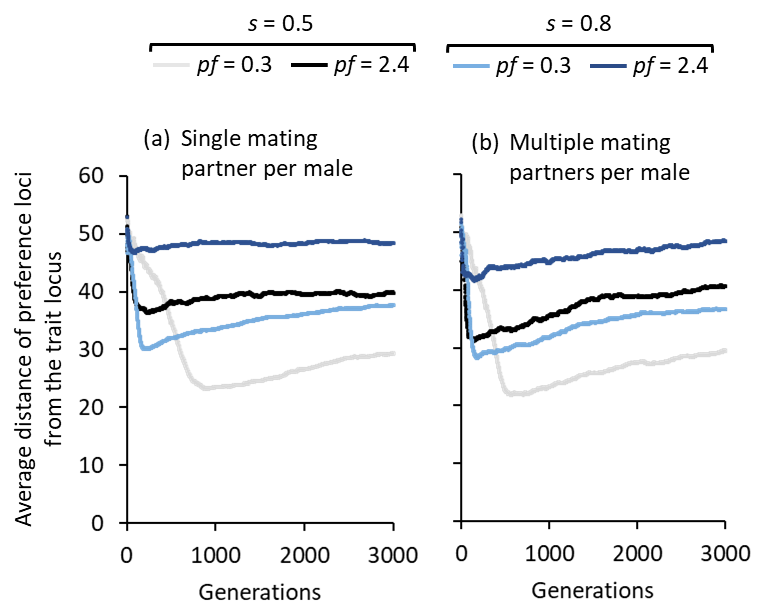


**Fig S3.1.** Changes in the level of physical linkage throughout 3000 simulated generations, when males are allowed to mate only once (a) or can mate with multiple females (b). Physical linkage is represented by the distance of mating preference loci from the trait locus. Results are presented for loci with alleles of preference for AA only, to prevent overlay, but are similar for loci with alleles of preference for A’A’. Simulation were run with a selection coefficient of *s*=0.5 (black and grey lines) or *s*=0.8 (dark and light blue lines), and with low and high preference strength factors (*pf*=0.3, grey and light blue lines; and *pf*=2.4, black and dark blue, respectively). For each of the four combinations average distances were calculated from 10 simulations.

Furthermore, allowing males to mate with multiple females did not improve population stability. Similar to the original model, one of the two homozygote phenotypes collapsed in the majority of simulations in which the selection coefficient was low (*s* = 0, 0.1, 0.2, 0.3, 0.4) or high (*s* = 0.9, 1). The proportion of unstable simulations for each low and high selection coefficient and low and high preference strength factor (*pf*), are detailed in the following table:

|  | *pf* = 0.3 | *pf* = 2.4 |
| --- | --- | --- |
| *s* = 0 | 16/20 | 17/20 |
| *s* = 0.1 | 20/20 | 20/20 |
| *s* = 0.2 | 20/20 | 20/20 |
| *s* = 0.3 | 20/20 | 15/20 |
| *s* = 0.4 | 20/20 | 0/20 |
| *s* = 0.9 | 20/20 | 11/20 |
| *s* = 1 | 20/20 | 20/20 |

**S3.2. Strength of preference towards the intermediate phenotype**

Since the heterozygotes (AA’) exhibit phenotypes that are intermediate to the two homozygote phenotypes (AA and A’A’), preference to mate with a heterozygote was originally assumed to be intermediate between the preference for each of the homozygote phenotypes. Therefore, if a female was paired with a heterozygote male, the probability to mate with him was a constant 0.5, regardless of the female’s mating preferences (Fig. 1).

Two alternative scenarios were tested, in which the choice of a mate based on its phenotype is either more flexible or less flexible, causing the attractiveness of intermediate phenotypes to be closer to that of the preferred or unpreferred phenotype, respectively. Accordingly, the strength of mating preference towards the heterozygote phenotype (AA’) was either increased or decreased. If preference was increased, the probability of a female to mate with a heterozygote male with which she was paired was halfway between 0.5 and the probability to mate with her preferred homozygote phenotype. If preference was decreased, the probability to mate with a heterozygote was halfway between 0.5 and the probability to mate with the unpreferred homozygote phenotype.

Increasing or decreasing the mating preference towards the intermediate phenotype had no influence on the general trends in simulation results. Physical linkage between trait and preference loci remained stronger for weaker preference strength and weaker selection pressure (Fig S3.2).


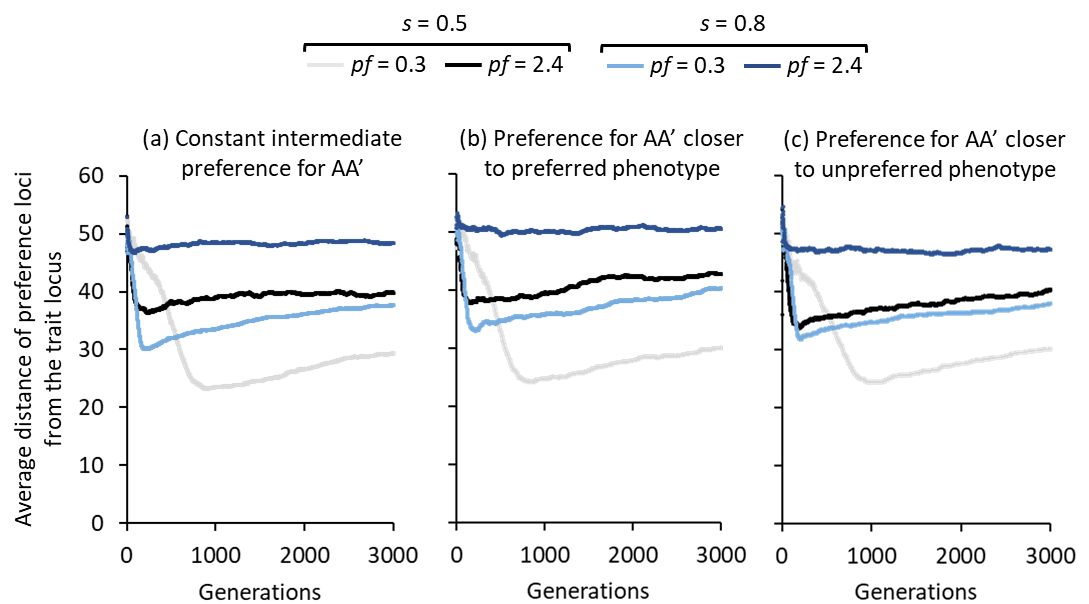


**Fig S3.2.** Changes in the level of physical linkage throughout 3000 simulated generations, assuming intermediate (a), elevated (b) or low (c) preference for heterozygotes, for individuals with a preference for one of the homozygote phenotypes. Physical linkage is represented by the distance of mating preference loci from the trait locus. Results are presented for loci with alleles of preference for AA only, to prevent overlay, but are similar for loci with alleles of preference for A’A’. Simulation were run with a selection coefficient of *s*=0.5 (black and grey lines) or *s*=0.8 (dark and light blue lines), and with low and high preference strength factors (*pf*=0.3, grey and light blue lines; and *pf*=2.4, black and dark blue, respectively). For each of the four combinations average distances were calculated from 10 simulations.

**S3.3 Number of recombination events per offspring batch**

In the original model, recombination occurs once in each parent and all four offspring inherit one of two possible haplotypes from each parent. Here we tested an alternative option in which separate recombination events take place for each offspring, simulating the formation of gametes with multiple possible haplotypes per parent. Recombination in this alternative form potentially shuffles haplotypes across the population more than in the original model. We therefore tested two recombination rates for this model version, with recombination occurring at a probability of either 1 or reduced to 0.5 per chromosome.

Adding additional recombination events per offspring batch did not influence the general trends in simulation results. Physical linkage between trait and preference loci remained stronger for weaker preference strength and weaker selection pressure (Fig. S3.3). However, since the total number of recombination events was increased across the population, the disruption of linkage disequilibrium was stronger compared to the original model. As a result, physical linkage was favoured even more and preference loci were closer to the ecological trait locus, on average, compared to the original model. In accordance, reducing the recombination rate, such that each chromosome recombines at a probability of 0.5, resulted in weakened physical linkage compared to simulations in which recombination occurred in all chromosomes (Fig. S3.3b,c). However, the influence of recombination rate on physical linkage is not surprising, since recombination is the force disrupting linkage disequilibrium between the ecological trait and mating preferences, and therefore, the force driving physical linkage. Similarly, physical linkage did not evolve when simulations of the original model were carried out without recombination, as a control.


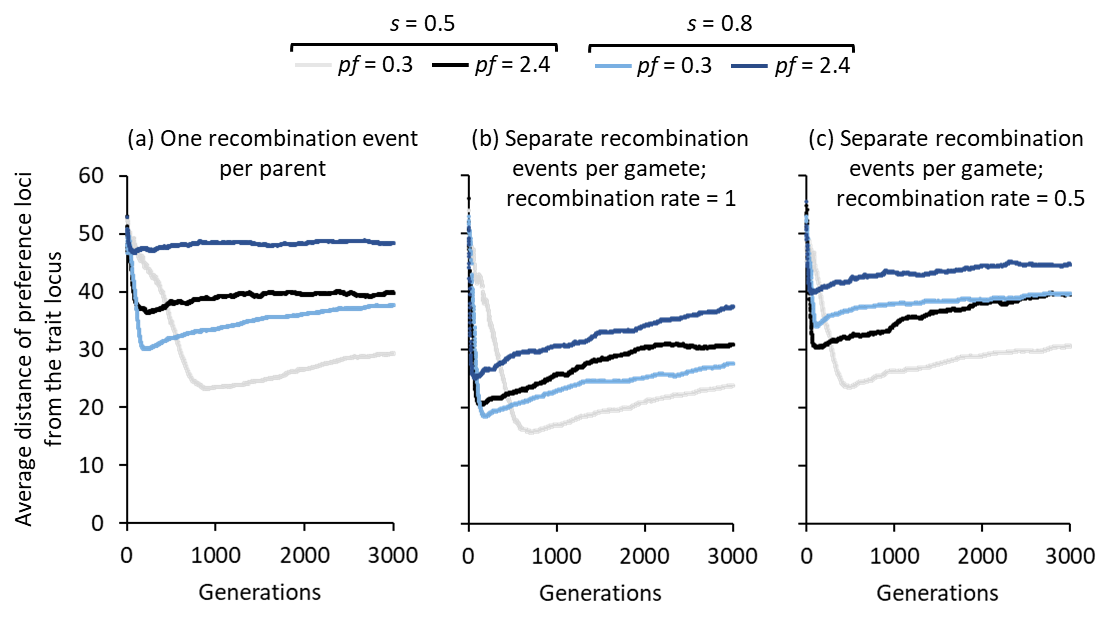


**Fig S3.3.** Changes in the level of physical linkage throughout 3000 simulated generations, when recombination occurs either once in each parent, resulting in two possible haplotypes across offspring of a single parent (a), or separately for each gamete, producing multiple possible haplotypes across offspring of a single parent (b,c). For the latter, recombination occurs either in every chromosome (b), or at a probability of 0.5 per chromosome (c). Physical linkage is represented by the distance of mating preference loci from the trait locus. Results are presented for loci with alleles of preference for AA only, to prevent overlay, but are similar for loci with alleles of preference for A’A’. Simulation were run with a selection coefficient of *s* = 0.5 (black and grey lines) or *s* = 0.8 (dark and light blue lines), and with low and high preference strength factors (*pf* = 0.3, grey and light blue; and *pf* = 2.4, black and dark blue, respectively). For each of the four combinations average distances were calculated from 10 simulations.

**S3.4 The level at which mutation rate is implemented**

In the original model, rather than mutations occurring at a certain probability at each locus, we simplified the implementation of mutations by randomly choosing 1 percent of individuals who received a mutation at a single randomly chosen locus within their “genome”. This was done to avoid iterating through all loci of all individuals, every generation. However, to examine whether the level at which mutation rate is implemented influences the model outcome, we tested an additional model version in which mutations occur at a probability of $5\cdot{10}^{-5}$ per single locus. The probability per locus was chosen to be equivalent to the calculated mutation rate per locus in the original model, in which mutations occur in 100 loci (1 percent of 10000 individuals) out of a total of 2 million loci in the entire population.

The level at which mutation was implemented in the model had no influence on the general trends in simulation results. Physical linkage between trait and preference loci remained stronger for weaker preference strength and weaker selection pressure (Fig. S3.4).


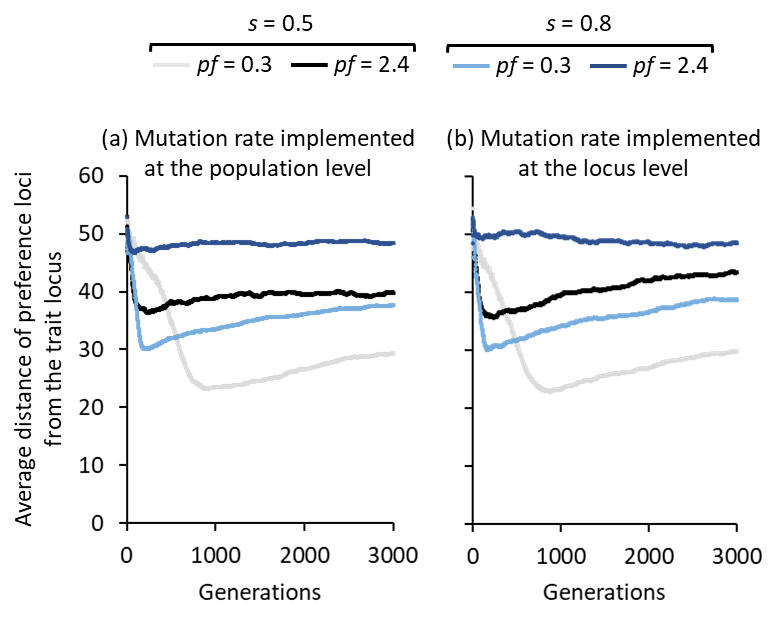


**Fig S3.4.** Changes in the level of physical linkage throughout 3000 simulated generations, when mutation rate is implemented either (a) at the population level (1% of individuals) or (b) at the single locus level (probability of $5\cdot{10}^{-5}$ per locus). Physical linkage is represented by the distance of mating preference loci from the trait locus. Results are presented for loci with alleles of preference for AA only, to prevent overlay, but are similar for loci with alleles of preference for A’A’. Simulation were run with a selection coefficient of *s* = 0.5 (black and grey lines) or *s* = 0.8 (dark and light blue lines), and with low or high preference strength factors (*pf* = 0.3, grey and light blue lines; and *pf* = 2.4, black and dark blue, respectively). For each of the four combinations average distances were calculated from 10 simulations.

**S3.5. Strength of selection against heterozygotes relative to homozygotes.**

In the original model we assumed selection acts equally against all phenotypes except AA in one habitat and A’A’ in the second habitat. In other words, the heterozygote phenotype (AA’) suffers from the same selection pressure as the unfavoured homozygote phenotype in each habitat. Here we test the influence of a more flexible approach, in which the heterozygote (AA’), intermediate, phenotype shares some of the favourable characters of each homozygote phenotype, causing it to be partially favoured in each habitat. Accordingly, the model was changed so that the selection force against heterozygotes was half of the selection force against homozygotes in the habitat in which they were not favoured. Three selection levels were tested: selection coefficients of *s* = 1, *s* = 0.9 and *s* = 0.8, for homozygotes in their unfavoured habitats, which were parallel to selection coefficients of *s* = 0.5, *s* = 0.45, and *s* = 0.4, for heterozygotes in both habitats. For lower selection coefficients, assortative mating was not fully established even after 3000 generations. For each selection level, results were tested for low and high preference strength factors (*pf* = 0.3 and *pf* = 2.4), with ten simulations each.

Physical linkage between ecological trait and mating preference loci remained stronger for weak compared to strong mating preferences. However, contrary to results of the original model, for low preference strength, weaker selection did not cause stronger physical linkage (Fig. S3.5). The positive correlation between physical linkage and selection force, for the low *pf* value, indicates that under these circumstances the direct influence of selection on physical linkage is stronger than its indirect influence via the strengthening of assortative mating. Most likely, this is the result of the very weak selection against heterozygotes compared to the original model.


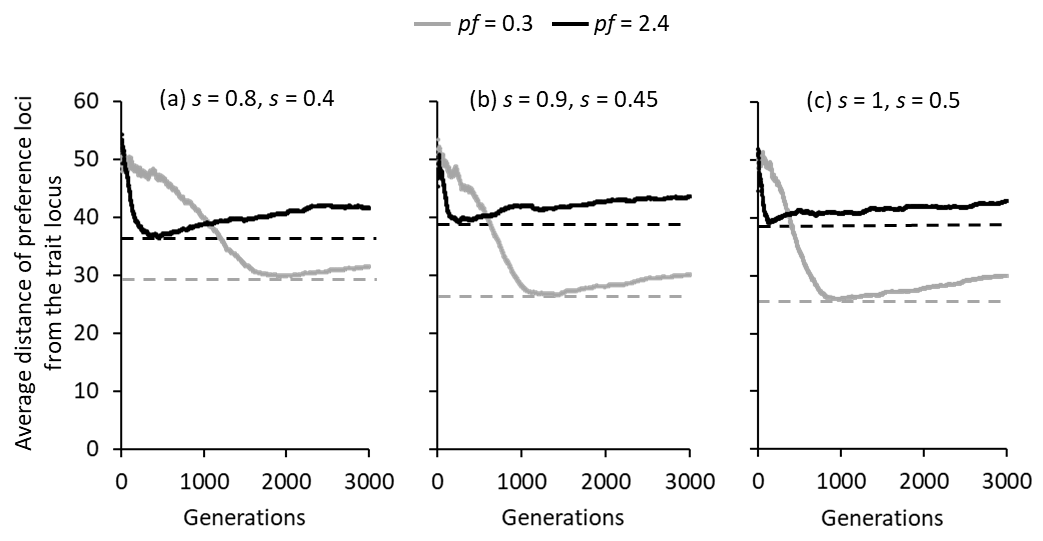


**Fig S3.5.** Changes in the level of physical linkage throughout 3000 simulated generations, assuming a selection coefficient (*s*) of 0.8, 0.9 or 1 for homozygote phenotypes in their unfavoured habitat, and respective intermediate selection coefficients of 0.4, 0.45 and 0.5 on heterozygote phenotypes (a, b and c, respectively). Physical linkage is represented by the distance of mating preference loci from the trait locus. Results are presented for loci with alleles of preference for AA only, to prevent overlay, but are similar for loci with alleles of preference for A’A’. Simulation were run with low and high preference strength factors (*pf* = 0.3, grey lines; and *pf* = 2.4, black lines, respectively). For each combination of selection coefficient and preference strength factor average distances were calculated from 10 simulations.

**S3.6. The number of loci controlling the ecological trait**

In the original model, the ecological trait is controlled by a single, diallelic locus. As an alternative, we developed a model in which the ecological trait is controlled by two separate loci, which arguably better reflects the genetic architecture of incompatibilities observed in hybrids between divergent taxa. The genetic basis of the trait in the model is similar to that of wing colour pattern in the two sympatric species *Heliconius melpomene* and *H. cydno*, where two major loci control red and white forewing bands respectively (Naisbit et al., 2003). To simplify the model description, we hereafter refer to colour pattern elements as the ecological trait, but the model could equally apply to a wide range of ecological traits. We note that similarly to the original one-locus model, the ecological trait in the modified two-locus model also serves as a mating cue (a “magic trait”, Gavrilets, 2004).

Differences compared to the original, one ecological trait locus model:

The two colour loci in this alternative model are located at opposite ends of the chromosome. The ‘Red’ locus is located at the first position within the chromosome and the ‘White’ locus is located at the last position within the chromosome. The colour loci each have two possible alleles which account for the presence (‘R’ and ‘W’ alleles) and absence (‘r’ and ‘w’ alleles) of a red and white band in the forewing, respectively. The alleles for presence of colour bands are dominant over those for absence. The presence of one colour band does not replace the presence of the other, resulting in a possible intermediate red-white band, characteristic of hybrids of *H. melpomene* and *H. cydno*. Together, there are nine possible genotypes at the two colour loci, and four possible colour phenotypes (Fig. S3.6.1b). The representations of genotypes and phenotypes in the model are based on descriptions of the genetic basis of forewing colour pattern in *H. melpomene*, *H. cydno* and their hybrids (Naisbit et al., 2003).


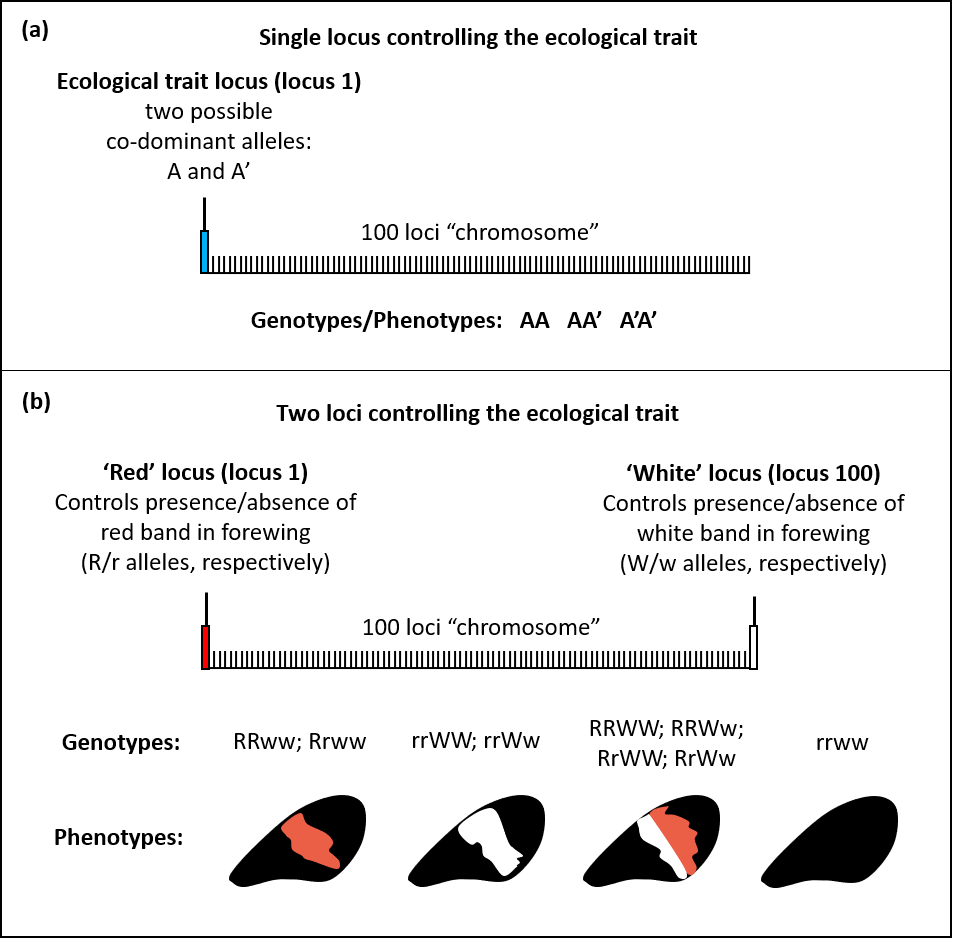


**Fig. S3.6.1.** Comparison of modeled “chromosomes” in the original model with one locus (a) versus two loci (b) controlling the ecological trait. We use wing colour pattern as an example to describe the ecological trait in the two-locus model, inspired by the two sympatric species *Heliconius melpomene* and *H. cydno*, characterised by a red and white/yellow forewing band, respectively. In the one-locus model, the single locus that controls the ecological trait is located at the first position within the “genome”. In the two-locus model, wing colour pattern is controlled by two biallelic loci, located at the first and last position within the “genome”. In both models individuals are diploid with a single pair of homologous chromosomes. In the one-locus model, the ecological trait locus alleles are codominant, resulting in three separate phenotypes: AA, A’A’ and an intermediate AA’ phenotype. In the two-locus model, the ‘R’ and ‘W’ alleles control the presence of a forewing red and white band, respectively, and are dominant, whereas the ‘r’ and ‘w’ alleles, for which each of the colours are absent, are recessive. The expression of one colour does not replace the expression of the other. Therefore, there are nine possible colour loci genotypes and four possible phenotypes: forewing red band, white band, red-white band and no-colour.

Similar to the original model, selection favours the red phenotype in one habitat and the white phenotype in the other, reflecting separate predators in each habitat, that learn to avoid different wing colour patterns (Mallet and Barton, 1989). The intermediate red-white phenotype and the no-colour phenotype are maladaptive in both habitats.

Randomly placed mutations cause mating preferences for either red or white phenotypes, similar to the preference for AA or A’A’ phenotypes in the model with one locus controlling the ecological trait. Neutral mutations, which do not contribute to mating preferences, are added as a reference. The probability of mating with an individual of red or white phenotype is dependent on the number of preference alleles and is calculated in the same way as in the model with one locus controlling the ecological trait (Fig. 1). All individuals exhibit an intermediate mating preference for the red-white and no-colour phenotypes with a constant mating probability of 0.5. All other processes were identical to the model with one locus controlling the ecological trait.

Because the changes we made in this alternative model are substantial compared to the original model, we tested the robustness of results across all analyses that were done on the original model. As there are two colour-loci, physical linkage between colour and preference loci was measured by recording the distance of every preference locus from the colour locus closest to it. Distances were then averaged per individual and then across individuals of the same simulation. To capture the locations of preference loci within the chromosomes, across the population, the positions of all preference loci in a single simulation were recorded, separately for chromosomes with presence of red (‘R’) and absence of white (‘w’) alleles, and for chromosomes with absence of red (‘r’) and presence of white (‘W’) alleles. The positions were recorded after 2000 generations, when the average distance of preference loci from the closest colour locus was found to be at a minimum across simulations.

The correlation between the average distance of preference loci from colour loci and the level of assortative mating was tested after 1500 generations, when variance in the proportion of phenotypically matching mating pairs across simulations was at a maximum in the model with two loci controlling the ecological trait.

Results of the alternative model with two loci controlling the ecological trait:

Although assortative mating developed at a slower rate in the one-locus model compared to the two-locus model, the overall trends were identical in both models. Similar to the one-locus model, in the two-locus model selection favoured genomes with preference loci adjacent to both colour loci. When physical linkage between colour and preference loci was strong, loci with alleles of preference for red were in proximity to both the presence of red and absence of white alleles in both colour loci (Fig. S3.6.2a). In accordance, loci with alleles of preference for white were in proximity to absence of red and presence of white allele Fig. S3.6.2b). No such linkage developed when no recombination was applied on offspring chromosomes, as a control (Fig. S3.6.2c,d).


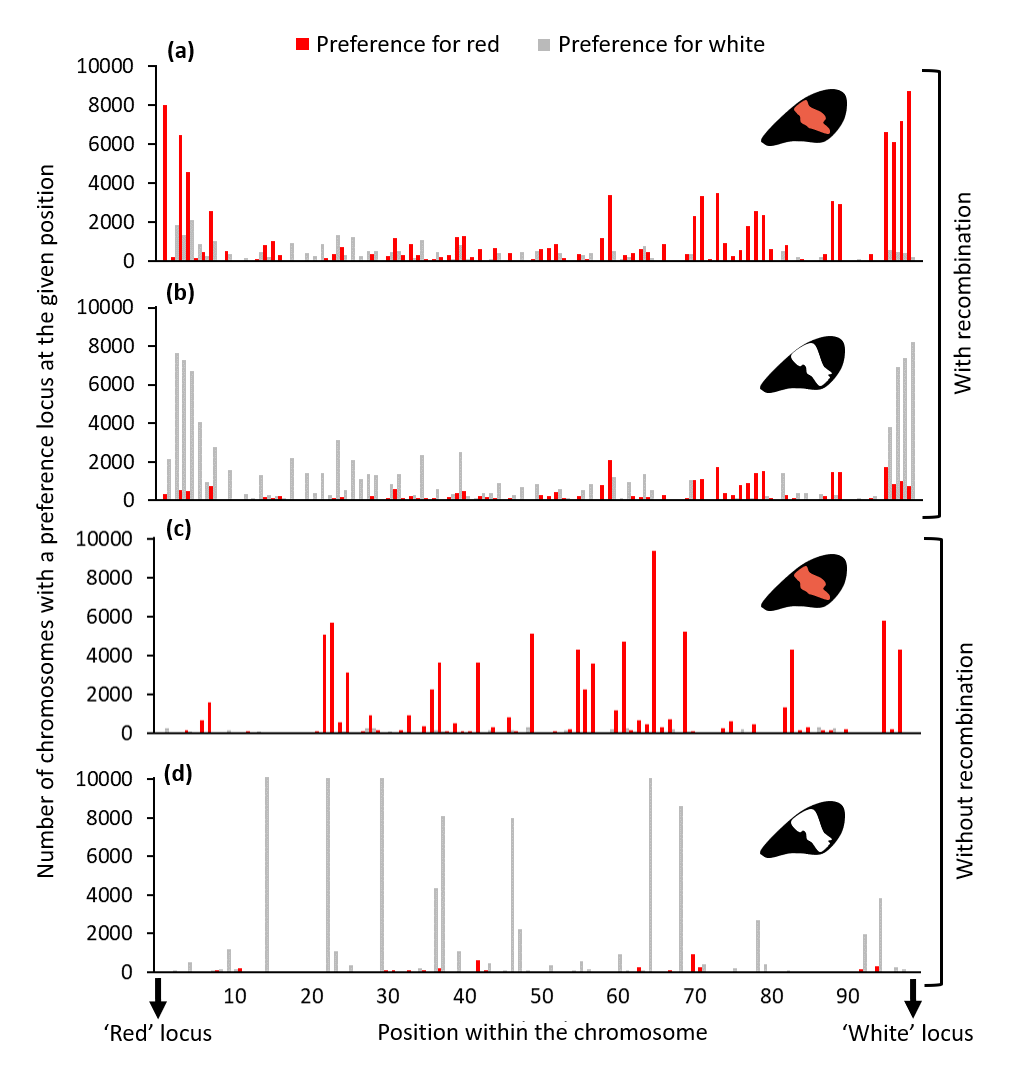


**Fig. S3.6.2.** Frequency of preference loci positions across the population (two-locus model). Data represent chromosomes of 10,000 individuals from a single example simulation with (a,b) and without (c,d) recombination in offspring chromosomes, with a selection coefficient of *s* = 0.5 and a mating preference strength factor of *pf* = 0.3 in both. The colour loci are placed at opposite ends of the chromosome, the ‘Red’ locus at the first position and the ‘White’ locus at the last. Loci positions are shown separately for chromosomes with presence of red and absence of white alleles (‘R’ and ‘w’, respectively; a,c) and those with absence of red and presence of white alleles (‘r’ and ‘W’, respectively; b,d). Positions were recorded after 2000 generations, when average distance of preference loci from the closest colour locus is minimal across simulations.

The influence of selection and mating preference strength on the development of physical linkage between colour loci and mating preference loci was the same as in the one-locus model. The minimum average distance between the colour loci and preference loci throughout the simulation was significantly smaller for weaker mating preference strengths (Fig. S3.6.3a,b, S3.6.4) and for lower selection coefficients (Fig. S3.6.4; *F_3,11_* = 5.5, *p* = 0.02 and *F_1,11_* = 70.8, *p* < 0.0001, respectively, for red preference, and *F_3,11_* = 3.8, *p* = 0.04 and *F_1,11_* = 61.3, *p* < 0.0001, respectively, for white preference). No significant interaction was found between selection and preference strength (*F_3,11_* = 1.0, *p* = 0.43 and *F_3,11_* = 0.1, *p* = 0.96, for red and white preference, respectively). Physical linkage did not develop between neutral loci and the colour loci (grey lines in Fig. S3.6.3a,b), nor between preference and colour loci in the absence of recombination (Fig. S3.6.3c).


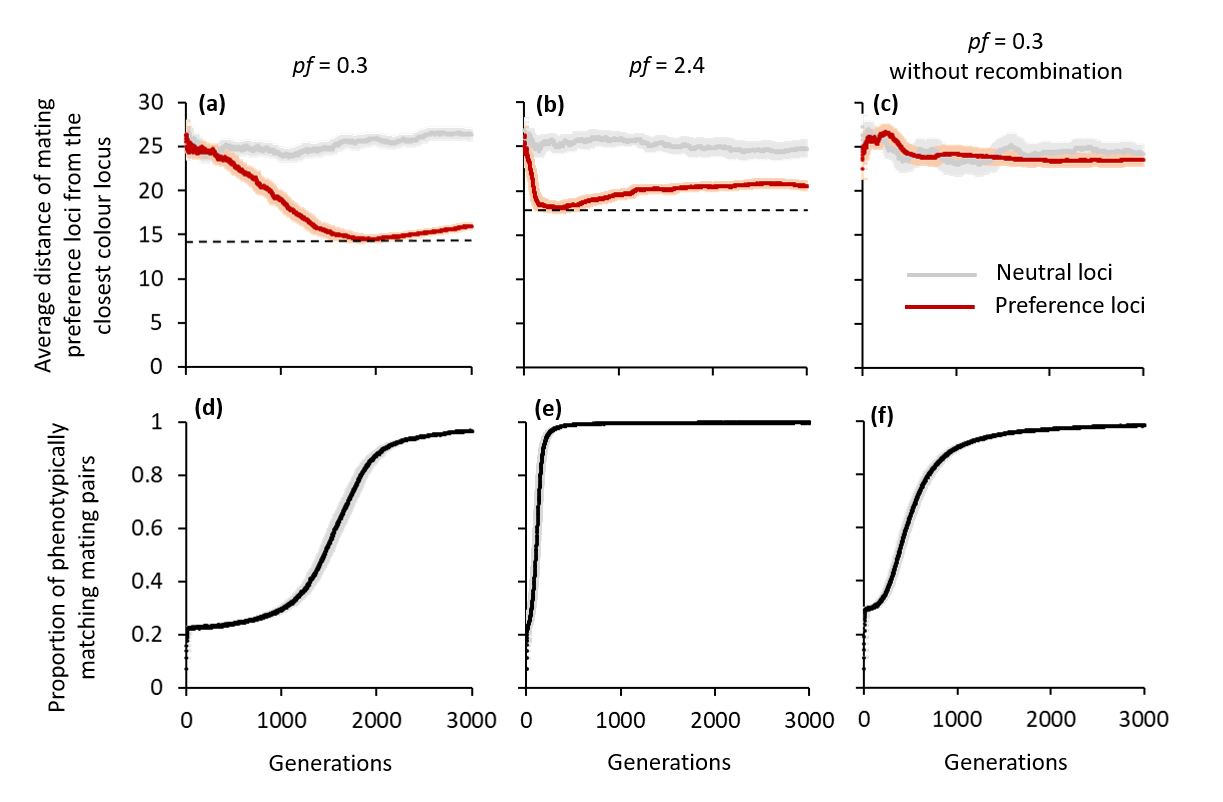


**Fig. S3.6.3.** Changes in the level of physical linkage (a,b,c) and assortative mating (d,e,f) throughout 3000 simulated generations (two-locus model). Physical linkage is represented by the distance of mating preference loci (red lines) and neutral loci (grey lines) from the closest colour locus. Results are presented for loci with alleles of preference for red only, to prevent overlay, but are similar for loci with alleles of preference for white. Horizontal dashed lines (a,b) mark the minimum average distance of preference loci from the closest colour locus. Simulation were run with a selection coefficient of s = 0.5, and with low and high preference strength factors (*pf* = 0.3, a,d; and *pf* = 2.4, b,e, respectively), and low *pf* with no recombination applied on offspring chromosomes, as a control (c,f). For each of the three, average distances and proportions were calculated from 20 simulations. Light-coloured shaded areas around the lines represent the Standard Error around the mean among simulations.


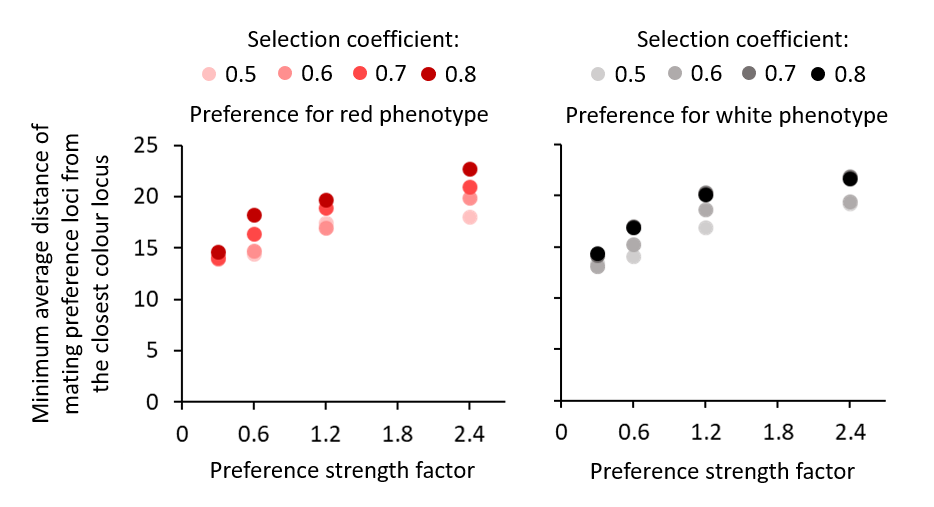


**Fig. S3.6.4.** Negative influence of selection strength and mating preference strength on physical linkage (two-locus model). Physical linkage is represented by the minimum average distance of mating preference loci from the closest colour locus, throughout 3000 simulated generations (marked with a dashed line in figures S2a and S2b). Minimum average values are based on 20 simulations for each parameter combination.

As in the one-locus model, significant negative correlations were found between the average distance of mating preference loci from the closest colour locus and the level of assortative mating (*r* = -0.55, *df* = 28, *p* = 0.002, and *r* = -0.66, *df* = 28, *p* < 0.001, for individuals of red and white phenotype, respectively; Fig. S3.6.5). Here too, no such correlation was found for neutral loci, with no influence on mating preference (*r* = -0.10, *df* = 28, *p* = 0.6, and *r* = -0.11, *df* = 28, *p* = 0.6, for individuals of red and white phenotype, respectively; Fig. S3.6.5).


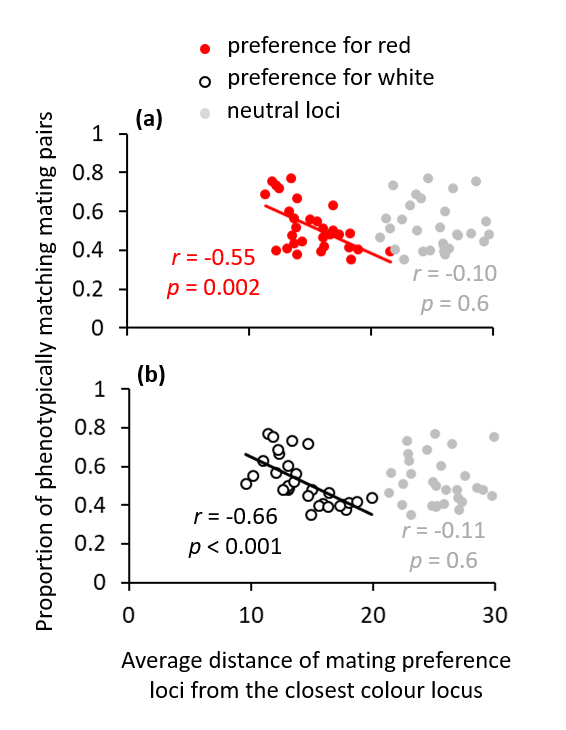


**Fig. S3.6.5.** Negative correlation between distance of preference loci from the colour loci and assortative mating (two-locus model). Data are presented separately for individuals of red (a) and white (b) phenotype, from 30 simulations with a selection coefficient of *s* = 0.5 and a mating preference strength factor of *pf* = 0.3. Values were recorded after 1500 generations, when variance in the proportion of phenotypically matching mating pairs across simulations was at a maximum.

Finally, we also tested whether locating the two colour loci on separate chromosomes, and therefore increasing the number of haplotypes that may arise, would have an influence on model results compared to locating both colour loci at opposite edges of a single chromosome, as described above. Similar to the original one locus model, each of the trait loci was placed at the first locus of each of the two separate chromosomes. The distance of preference loci from the trait locus was averaged across both pairs of homologous chromosomes (four chromosomes in total) per individual. As in all simulations, the distance was then averaged across all individuals in the population, each generation. Ten simulations were carried out for each combination of the lowest and highest preference strength factors (*pf* = 0.3 and *pf* = 2.4) and selection coefficients (*s* = 0.5 and *s* = 0.8) that were tested in the original model. Trends were then compared to the original model in which the two ecological trait loci were placed on a single chromosome.

Placing the loci on separate chromosomes had no influence on the general trends, i.e. physical linkage between ecological trait and mating preference loci remained stronger for weaker mating preferences and weaker selection strength (Fig. S3.6.6).

**
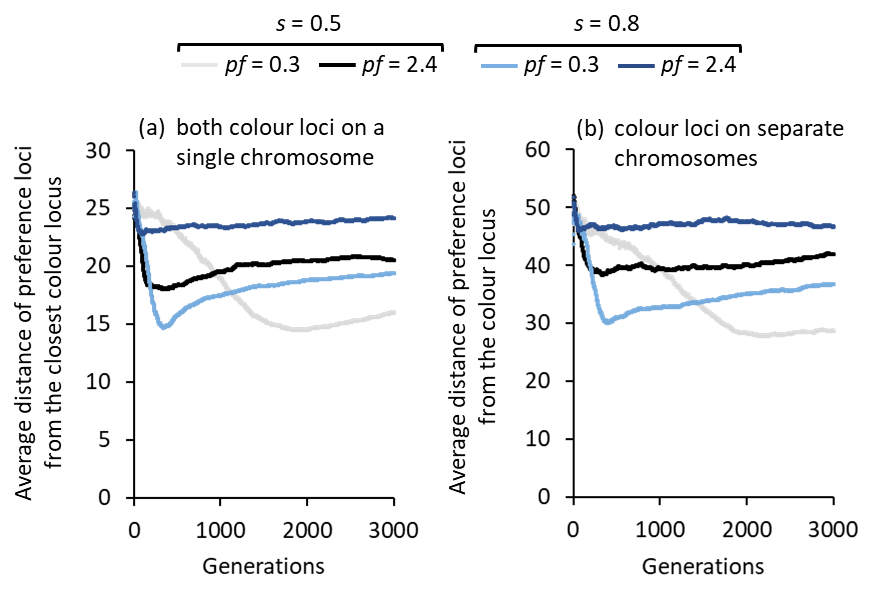
**

**Fig S3.6.6.** Changes in the level of physical linkage throughout 3000 simulated generations, for a model in which both colour loci are located at opposite ends of a single chromosome (a) or on separate chromosomes (b). Physical linkage is represented by the distance of mating preference loci from the colour locus. Results are presented for loci with alleles of preference for red only, to prevent overlay, but are similar for loci with alleles of preference for white. Simulation were run with a selection coefficient of *s*=0.5 (black and grey lines) or *s*=0.8 (dark and light blue lines), and with low and high preference strength factors (*pf*=0.3, grey and light blue lines; and *pf*=2.4, black and dark blue, respectively). For each of the four combinations average distances were calculated from 10 simulations.

**References**

Naisbit, R. E., C. D. Jiggins, and J. Mallet. 2003. Mimicry: developmental genes that contribute to speciation. Evolution and Development 5:269-280.

Mallet, J., and N. H. Barton. 1989. Strong natural-selection in a warning color hybrid zone. Evolution 43:421-431.
