# Supplementary material for: Evolution of physical linkage between loci controlling ecological traits and mating preferences
Source: J Evol Biol. 2022 Oct 5;35(11):1537–47. doi: 10.1111/jeb.14105 (PMC9827829; doi:10.1111/jeb.14105)

**Appendix S4. Supplementary data figures**

**Figure S4.1.** Changes in average phenotype abundance throughout 3000 simulated generations. Simulations were run with a selection coefficient of *s*=0.5, and with low (a) and high (b) preference strength factors (*pf* = 0.3 and *pf* = 2.4, respectively). The AA phenotype abundance values overlap those of the A’A’ phenotype abundance and are therefore hidden. Average phenotype abundances were calculated from 20 simulations. The Standard Error around the mean among simulations is represented by the grey, light grey and light blue shaded areas around the AA, AA’ and A’A’ abundance lines, respectively.


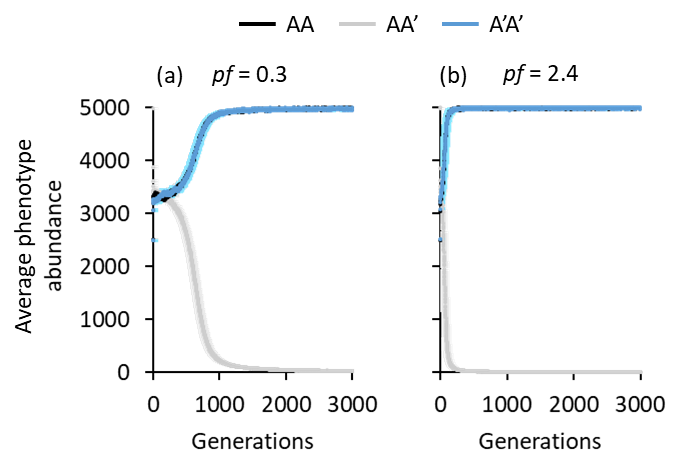


**Figure S4.2.** Changes in average number of preference loci with AA preference alleles (black lines), A’A’ preference alleles (blue lines) and neutral alleles in the genomes of AA phenotyped individuals (a,b) and A’A’ phenotyped individuals (c,d) throughout 3000 simulated generations. Simulations were run with a selection coefficient of *s*=0.5, and with low (a,c) and high (b,d) preference strength factors (*pf* = 0.3 and *pf* = 2.4, respectively). Average loci numbers were calculated from 20 simulations. The Standard Error around the mean among simulations is represented by the grey, light blue and light grey shaded areas around the AA preference, A’A’ preference and neutral allele lines, respectively.


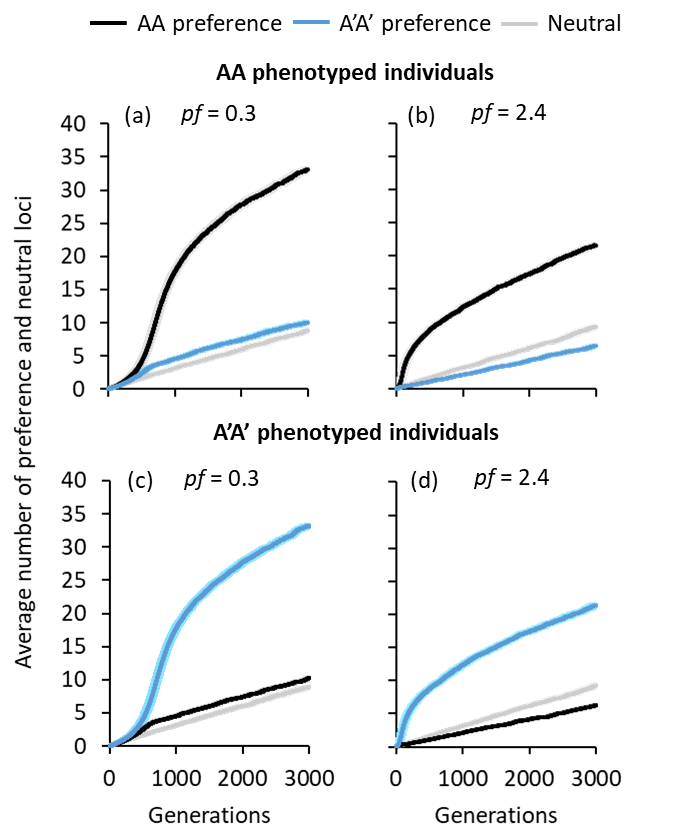


**Figure S4.3.** Changes in average probability of AA phenotyped females (a,b) and A’A’ phenotyped females (c,d) to mate with males of each of the homozygote phenotypes throughout 3000 simulated generations. Simulations were run with a selection coefficient of *s*=0.5, and with low (a,c) and high (b,d) preference strength factors (*pf* = 0.3 and *pf* = 2.4, respectively). Average probabilities to mate were calculated from 20 simulations. The Standard Error around the mean among simulations is represented by the grey and light blue shaded areas around the probability to mate with AA and A’A’, respectively.


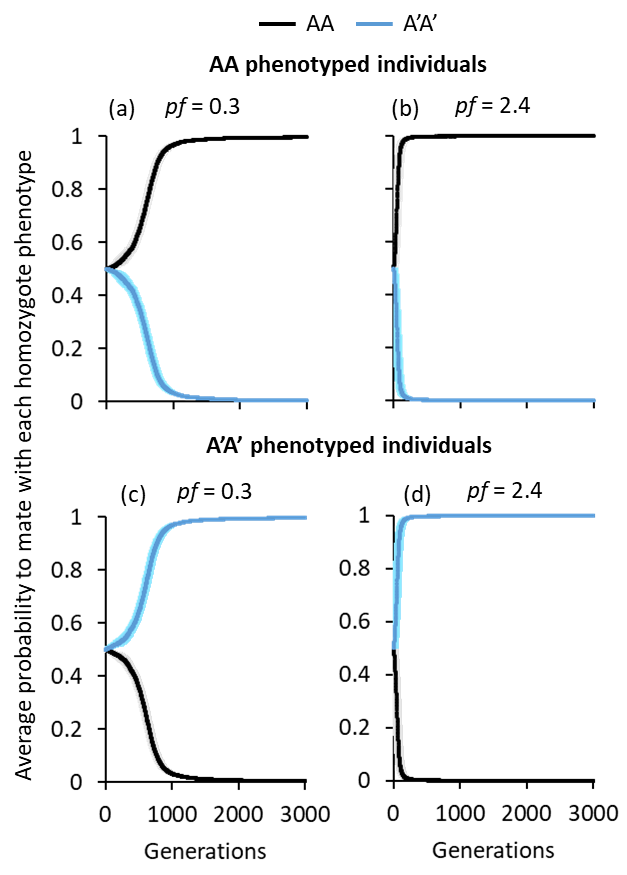

Supplement: Supplementary file 4 — Appendix S4 [file JEB-35-1537-s001.docx]
